# Size and sequence variation of the Lid of the Cas12a nuclease domain results in a nickase phenotype

**DOI:** 10.1093/nar/gkag934

**Published:** 2026-10-05

**Authors:** Ricardo Villegas Warren, Satoshi N Omura, Diana Leon Castro, Christian Südfeld, Maartje J Luteijn, Hayato Morinaga, Wakana J Inoue, Yutaro Shuto, Anne-Marie Kuijpers, Frank Meulewaeter, David De Vleesschauwer, Raymond H J Staals, Osamu Nureki, Wen Y Wu, John van der Oost

**Affiliations:** Laboratory of Microbiology, Wageningen University & Research, Stippeneng 4, 6708 WE Wageningen, Netherlands; Department of Biological Sciences, Graduate School of Science, University of Tokyo, Tokyo 113-0033, Japan; Laboratory of Microbiology, Wageningen University & Research, Stippeneng 4, 6708 WE Wageningen, Netherlands; Laboratory of Microbiology, Wageningen University & Research, Stippeneng 4, 6708 WE Wageningen, Netherlands; Laboratory of Microbiology, Wageningen University & Research, Stippeneng 4, 6708 WE Wageningen, Netherlands; Department of Biological Sciences, Graduate School of Science, University of Tokyo, Tokyo 113-0033, Japan; Department of Biological Sciences, Graduate School of Science, University of Tokyo, Tokyo 113-0033, Japan; Department of Biological Sciences, Graduate School of Science, University of Tokyo, Tokyo 113-0033, Japan; BASF Belgium—Innovation Center Gent, Technologiepark-Zwijnaarde 101, 9052 Ghent, Belgium; BASF Belgium—Innovation Center Gent, Technologiepark-Zwijnaarde 101, 9052 Ghent, Belgium; BASF Belgium—Innovation Center Gent, Technologiepark-Zwijnaarde 101, 9052 Ghent, Belgium; Laboratory of Microbiology, Wageningen University & Research, Stippeneng 4, 6708 WE Wageningen, Netherlands; Department of Biological Sciences, Graduate School of Science, University of Tokyo, Tokyo 113-0033, Japan; Laboratory of Microbiology, Wageningen University & Research, Stippeneng 4, 6708 WE Wageningen, Netherlands; Laboratory of Microbiology, Wageningen University & Research, Stippeneng 4, 6708 WE Wageningen, Netherlands

## Abstract

CRISPR–Cas12a nucleases typically induce double-strand breaks (DSBs) to initiate genome editing. However, reliance on DSBs risks chromosomal rearrangements and unintended insertions or deletions at off-target sites. Therefore, engineering nickase variants that cleave only a single DNA strand might enable precise editing platforms. We describe an unbiased *in vivo* screen in *Escherichia coli* to identify LbCas12a nickase variants. We targeted the RuvC Lid domain for mutagenesis, hypothesizing that altering this region would disrupt the coordination of strand cleavage. While the screen produced many nickase variants with targeted point substitutions (Lid1), it also serendipitously yielded a variant (Lid2) with an unprecedented Lid domain in terms of size and sequence. Biochemical characterization confirms that Lid2 robustly nicks the non-target strand (NTS) with minor cleavage activity observed for the target strand (TS), accumulating predominantly nicked substrates. Cryo-electron microscopy (cryo-EM) at 2.5 Å resolution reveals that, in Lid2, the native α-helical Lid is replaced, uncoupling the concerted cleavage mechanism. Specifically, the remodeled Lid appears to stabilize the NTS in the active site, whereas it hampers the conformational transitions required for subsequent TS cleavage. This robust nickase-phenotype of this Cas12a variant expands the CRISPR–Cas toolbox and offers a new component for precise genome editing applications.

## Graphical Abstract

**Graphical Abstract:**
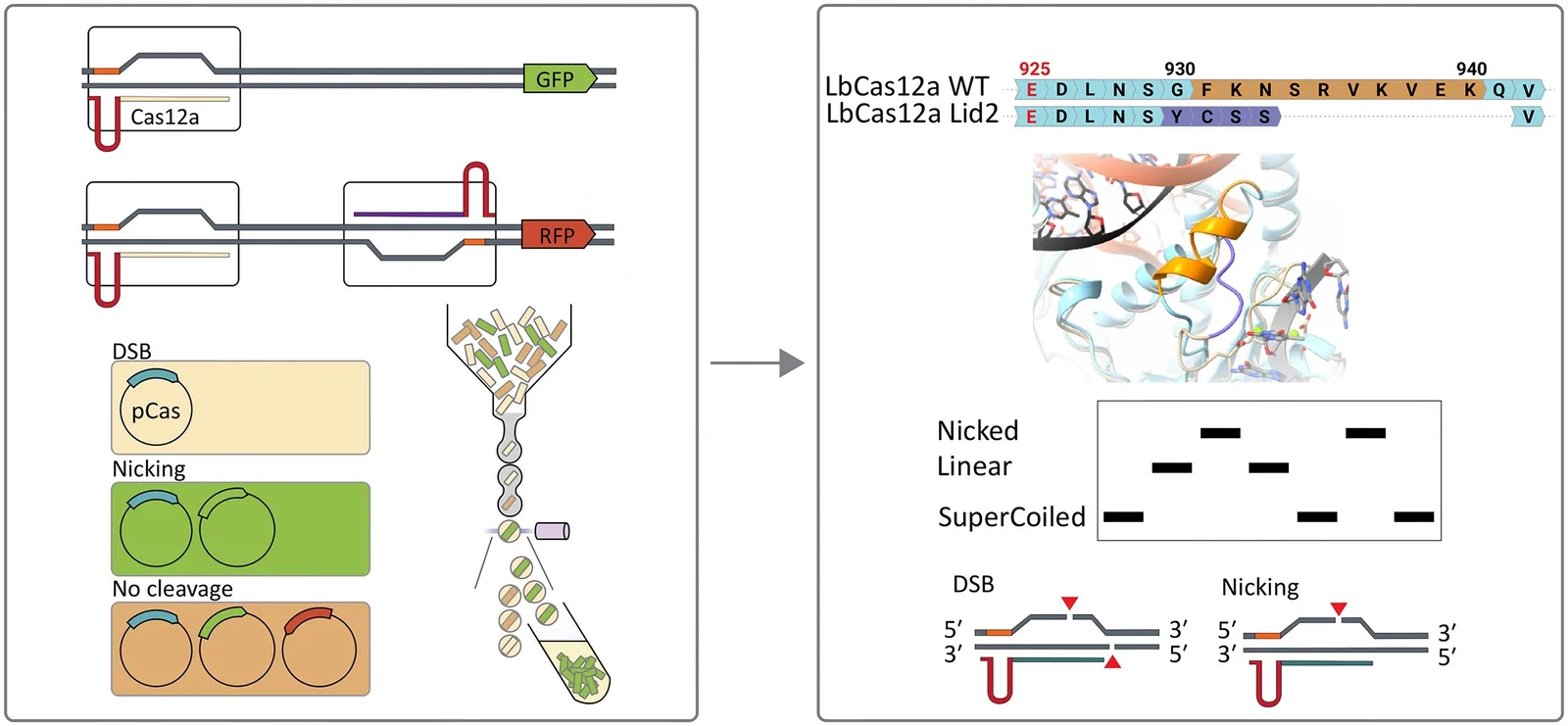

## Introduction

CRISPR–Cas systems provide bacteria and archaea with an adaptive, heritable defense against bacteriophages and other mobile genetic elements (MGEs) [1–4]. Genomic CRISPR loci capture invader-derived spacers that are transcribed and processed into CRISPR RNAs (crRNAs) that assemble with Cas effector proteins to direct sequence-specific cleavage of complementary nucleic acids [1, 2]. The ease of adjusting the target specificity of these RNA-guided nucleases has transformed CRISPR–Cas into a versatile platform for genome manipulation [5, 6]. However, most widely used effectors generate double-strand breaks (DSBs) that may result in undesired editing outcomes [7–9]. To improve their precision and to avoid genotoxicity and enable editing modalities, Cas effectors have been converted into nickases, which generate single-strand breaks (SSBs) [7, 10].

Two classes of DNA-targeting CRISPR–Cas systems have been defined [5, 11]: Class 1 systems, which utilize multimeric protein complexes as effectors for interference and degradation of the targeted genetic material, and Class 2 systems, in which recognition and cleavage of the target sequences are carried out by a single multi-domain effector protein [6, 9]. The best characterized Class 2 nucleases are Cas9 and Cas12a, which differ substantially with respect to their mechanisms of recognizing and cleaving target DNA [7]. For instance, as a first step in the target search, Cas9 recognizes a G-rich protospacer adjacent motif (PAM) located downstream of the target sequence (protospacer), whereas Cas12a recognizes a T-rich PAM positioned upstream of the protospacer [12]. Moreover, as the final step of dsDNA target interference, Cas9 employs two distinct nuclease domains: the RuvC domain cleaves the displaced non-target strand (NTS), while the HNH domain cleaves the guide-bound target strand (TS), thereby generating blunt-ended DSBs [7, 13]. By contrast, Cas12a uses a single RuvC active site to cleave both DNA strands and produce staggered DSBs, with overhangs of 2–10 nucleotides [12, 14]. Early crystallographic snapshots of Cas12a led to the proposal of a putative second nuclease (“Nuc”) site within the RuvC–Nuc lobe that might mediate TS cleavage [15]. However, around the same time a structural analysis was performed on another Cas12 subtype (C2c1, later called Cas12b) that revealed that both the TS and the NTS are being bound (and cleaved) independently by the single RuvC catalytic pocket [16]. Later biochemical and structural studies on Cas12a demonstrated that also this nuclease relies exclusively on the single RuvC active site for cleavage of both strands, with the sequential cleavage of NTS and TS [6, 17], although many of the related mechanistic details remained elusive. On the one hand these basic mechanistic distinctions between Cas9 and Cas12a reflect the divergent evolution of related transposon-associated nuclease ancestors toward distinct prokaryotic immune systems, and on the other hand they have important implications for repurposing them for complementary genome editing applications [5, 9, 18, 19].

After elucidation of many structural and functional features, Cas9 and Cas12 proteins have been harnessed as highly efficient genome-editing tools in a wide range of cells and organisms [9, 20, 21]. When introduced in cells of interest, both nucleases generate targeted DSBs that then generally trigger endogenous DNA repair pathways of host cells: either error-prone non-homologous end joining (NHEJ) or microhomology-mediated end joining (MMEJ) pathways that results either in insertions or deletions (indels) or homology-directed repair (HDR) that relies on supplemented homologous DNA templates to generate precise edits [20, 22–24]. The efficiency of HDR, however, not only varies widely across species and cell types but also depends strongly on the design of the repair fragment [23, 25].

Given that CRISPR–Cas systems evolved as a defense mechanism, one would expect some tolerance for mismatches in the target sequences as that would still allow for recognition of rapidly evolving MGEs that might otherwise escape detection [4]. In a biotechnological context for genome editing, however, specificity is key to avoid introducing undesired DSBs at off-target sites that share partial homology with the target sequence [26, 27]. One way to reduce off-target editing is to engineer a Cas9 nickase by inactivating one of the two active site domains, either RuvC that catalyzes cleavage of the NTS or HNH that is responsible for cleaving the TS. As such, the generation of a DSB requires a nickase pair that is guided by two different RNA guides that each introduce a nick in opposing strands of two adjacent targets, thereby increasing the specificity of genome editing [7, 10, 20]. The paired-nickase method contributes to increased specificity, but it does not eliminate the risk of undesired chromosomal rearrangements, as it still results in a DSB formation [20, 27]. Therefore, strategies have been developed to avoid DSBs by harnessing nickase variants of Cas nucleases fused to other effector domains that modify DNA sequences [28, 29, 30]. A first example of a CRISPR-based editing tool that does not require a DSB is the Base Editor (BE), which uses a Cas9 nickase (nCas9, RuvC inactivated, i.e. nicking elying of TS) fused to a deaminase domain (C-to-T, or A-to-G) [28, 29]. While the nick is not strictly required for base conversion, introducing a SSB in the non-edited strand enhances editing efficiency [29]. After binding a dsDNA target, these nCas9-BEs nick the TS, while one or more nucleotide bases of the NTS are deaminated [28, 29]. Subsequently, the nick in the non-edited TS stimulates the mismatch repair (MMR) pathway, which promotes repair using the modified, base-edited strand as a template, resulting in the desired edit [28–30].

A second editing strategy that avoids DSB formation is the development of nickase-based prime editors, which use nCas9 (HNH inactivated) in combination with a reverse transcriptase (RT). Prime editors require a prime editing guide RNA (pegRNA) that not only consists of a 5′ guide sequence that binds the TS but also a central RT template (RTT) that includes the desired editing information, and a 3′ primer binding site (PBS) that allows binding to the complementary displaced NTS. After binding and nicking of the NTS, the PBS part of the pegRNA forms a heteroduplex with the complementary cleaved DNA product, after which the RT catalyzes elongation, resulting in the desired RTT-encoded edits. In this way, substitutions, insertions, and deletions can specifically be introduced [31, 32].

Due to its earlier discovery, Cas9 is the most frequently used Cas protein for genome editing [33]. During the past decade, however, Cas12a has also been broadly adapted for genome-editing applications in eukaryotic cells [9, 18]. As described above, Cas12a differs from Cas9 in that it lacks an HNH nuclease domain; hence, it solely relies on its single RuvC active site to cleave both DNA strands [17]. DSB generation by Cas12a proceeds through a multi-step mechanism that starts with PAM recognition and local DNA unwinding, followed by inspection of guide–target complementarity and extension of the R-loop [15, 34]. These events drive conformational rearrangements in the REC lobe and the RuvC Lid sub-domain, which activate the RuvC catalytic center for DNA scission [35, 36]. Although observed in some initial crystal structures reported by Yamano *et al*. [15, 34], the RuvC Lid sub-domain was first defined as a discrete structural element by Stella *et al*. [37]. More recently, the α-helical Lid was demonstrated to guide the target DNA toward the RuvC active site [38]. Once activated, Cas12a cleaves DNA in a strictly sequential manner, first attacking the NTS and subsequently the TS using the same RuvC active site [17].

Because Cas12a nucleases have a single nuclease domain (RuvC) that cuts both strands, it is not straightforward to engineer a nickase [6]. Still, some amino acid substitution variants of Cas12a have recently been reported to have nickase activity due to reduced TS cleavage activity. However, these variants often exhibit significantly reduced catalytic efficiency or residual double-strand cleavage activity [21, 34, 39–41]. Because DSB cleavage by Cas12a occurs in two steps, we hypothesized that by combining rational design and random mutagenesis, it should be possible to modify Cas12a into a variant with a robust nickase phenotype that predominantly cuts only the NTS by disrupting some of the interactions that coordinate the transition toward cleavage of the TS. In this study, we describe the generation and characterization of many novel LbCas12a nickase variants, one of which (Lid2) was selected for in-depth molecular characterization. Integration of our data with recently published structural and molecular dynamics analyses has resolved some key details of the Cas12a two-step catalytic mechanism, explaining how the Lid2 variant uncouples strand cleavage.

## Materials and methods

### Bacterial strains and growth conditions

All *in vivo* screening experiments were performed in *Escherichia coli* DH10B cells (Thermo Fisher). Cells were cultured in LB medium at 37°C with appropriate antibiotics (chloramphenicol, kanamycin, or ampicillin) for plasmid selection. For protein expression, His₆-tagged LbCas12a variants were produced in *E. coli* Rosetta (DE3) strains grown in LB at 37°C and induced with isopropyl β-D-1-thiogalactopyranoside (IPTG) at mid-log phase, followed by overnight expression at reduced temperature. Rosetta strains were maintained with chloramphenicol alongside the expression plasmid antibiotic.

### *In vivo* FACS-based nicking screen of the LbCas12a Lid mutant library

A saturation mutagenesis library targeting residues 931–940 of the Lid domain in LbCas12a was constructed using Golden Gate assembly (see *LbCas12a Saturation Mutagenesis Library Construction*) and transformed into *E. coli* DH10B cells harboring two reporter plasmids, pGFP and pRFP. These reporters contain distinct Cas12a target sites under constitutive promoters, enabling discrimination between wild-type (GFP⁻/RFP⁻), dCas12a (GFP⁺/RFP⁺), and nickase (GFP⁺/RFP⁻) phenotypes. Library-transformed cells were grown overnight in LB supplemented with chloramphenicol (25 µg/ml), diluted 1:100 into fresh medium, and grown to mid-log phase (OD₆₀₀ ∼0.4–0.6). Cells were harvested, washed in PBS, and subjected to fluorescence-activated cell sorting (FACS) on a Sony SH800S equipped with 488 and 561 nm lasers. Sorting gates were established using wild-type and dCas12a controls. GFP⁺/RFP⁻ cells were collected into LB–chloramphenicol, recovered for 1 h at 37°C, and plated. Colonies were screened by fluorescence, pooled, and subjected to a second round of enrichment. Plasmids from selected clones were isolated and sequenced (Macrogen Europe) to identify Lid mutations.

### Quantification and statistical analysis

Flow cytometry data were analyzed using Sony SH800S software. Gating thresholds were defined using wild-type and dCas12a controls. Enrichment relied on qualitative GFP⁺/RFP⁻ phenotypes. No statistical tests were applied.

### Recombinant DNA

#### Plasmid preparation

Plasmids were cloned in *E. coli* DH10B chemically competent cells (single-genotype constructs) or One Shot TOP10 Electrocomp cells (Thermo Fisher) for library construction. PCR amplification was performed using Q5 High-Fidelity DNA Polymerase (NEB), analyzed by agarose gel electrophoresis, and gel-purified (Zymoclean Gel DNA Recovery Kit, D4002). Primers <60 bp were synthesized by IDT; longer oligos and gBlocks were obtained from Sigma–Aldrich. Initial validation was performed by Sanger sequencing (Macrogen), and full-length plasmids were confirmed by Oxford Nanopore sequencing (Plasmidsaurus).

#### Construction of Pgfp and Prfp reporter plasmids

The reporter-plasmid backbones (pGFP, pSC101 origin; pRFP, pBR322 origin) were adapted from the pCas/pCRISPR/pTargetF CRISPR–Cas9 editing plasmids [42, 43]. Reporter plasmids for *in vivo* cleavage assays were generated in a two-step cloning workflow. For pGFP, protospacer PS1 was inserted by annealing BG24541/BG24542, digesting with KpnI/AatII, and ligating into the GFP backbone. pRFP was engineered to contain two adjacent protospacers (PS1 and PS2, separated by 24 bp) by BamHI/AatII digestion and ligation of gBlock BG24658. Constructs were transformed into DH10B and selected on kanamycin (pGFP) or ampicillin (pRFP). Correct clones were verified by Sanger sequencing. Primer and spacer sequences appear in Supplementary Tables S1–S3. Maps of both reporter plasmids are provided in Supplementary Fig. S9.

#### Incorporation of crRNA guides into reporter plasmids

crRNA cassettes were introduced by Golden Gate assembly. PCR fragments containing Esp3I (pGFP) or BbsI (pRFP) sites were amplified using primers encoding the LbCas12a direct repeat (BG25639, BG25644) and the PJ23119 promoter (BG25640, BG25643). Gel-purified fragments were assembled with annealed spacer oligos (Spacer1 for pGFP; Spacer2 for pRFP) using Esp3I or BbsI and T4 DNA Ligase, transformed into DH10B, and sequence-verified. Primer and spacer sequences appear in Supplementary Tables S1–S3.

#### Site-directed mutagenesis of LbCas12a and SpCas9

Single-genotype variants were generated using SapI-based Golden Gate mutagenesis. SapI-flanked PCR fragments were amplified from wild-type plasmids (pRV060 for LbCas12a or the SpCas9 expression vector). Mutagenic oligonucleotides were annealed in CutSmart buffer and assembled with ∼100 ng PCR product using SapI and T4 DNA Ligase. Ligated DNA was purified (Zymoclean DNA Clean & Concentrator, D4004), transformed into DH10B or TOP10, and confirmed by Sanger sequencing. Templates and oligonucleotides are listed in Supplementary Tables S1–S3.

#### LbCas12a saturation mutagenesis library construction

To generate a saturation mutagenesis library covering residues 931–940 of the Lid domain, 10 consecutive NNK codons were introduced by Golden Gate assembly. A SapI-flanked backbone fragment was PCR-amplified from pRV060 using primers BG25788/BG25789. Complementary oligos BG25836/BG25837 encoding the NNK block were annealed and assembled with the backbone using SapI and T4 DNA Ligase. Purified products were electroporated into *E. coli* DH5α or TOP10, recovered, plated on chloramphenicol, and pooled.

#### Lid trimming

Plasmids derived from LbCas12a wild-type (Lid1Δ series) and LbCas12a Lid2 (Lid2Δ series) were constructed, each carrying a defined deletion within the Lid region. In Lid1Δ variants amino acid residues 930–941 are progressively trimmed, deleting 1–12 residues either from the C-terminal end of the Lid (Lid1Δ1-C to Lid1Δ12-C) or from its N-terminal end (Lid1Δ1-N to Lid1Δ12-N). In addition, the 1–4 residues of the Lid2 variant (YCSS) are deleted from the C-terminal end (Lid2Δ1-C to Lid2Δ4-C); details are described in Supplementary Table S5. Each plasmid was assembled by back-to-back PCR, followed by blunt-end ligation, using a common 5′-phosphorylated reverse primer and a forward primer that introduces the variant-specific deletion; the used oligonucleotides are listed in Supplementary Table S6. PCR products were treated with DpnI, checked on 1% agarose gels, purified (Zymoclean DNA Clean & Concentrator, D4004), and ligated using approximately 75 ng of purified product in 10 µl reactions with T4 DNA Ligase. Half of each ligation was transformed into *E. coli* DH10B and confirmed by Sanger sequencing.

#### Lid trimming assay

All confirmed trimming-series plasmids, together with the wild-type LbCas12a, the catalytic-dead E925A/D1180A control and the parental Lid2 reference, were diluted to 10 ng/µl and transformed in triplicate using 30 ng of plasmid and 50 µl of *E. coli* DH10B cells harboring the pGFP and pRFP reporters Three biological replicates each one with three technical replicates were performed. Cells were recovered for 1 h at 37°C and 180 rpm in a 96-well, 2 ml Master Block (Greiner Bio-One, 780 271) containing 200 µl of LB medium. Recovered cultures were diluted 1:10 into a fresh Master Block (Greiner Bio-One, 780271) containing M9TG medium [M9 minimal salts (KH_2_PO_4_ 22 mM, NaCl 8.56 mM, Na_2_HPO_4_ 47.8 mM, NH_4_Cl 18.7 mM), 1% (w/v) tryptone, and 54.3 mM glycerol] supplemented with chloramphenicol (25 µg/ml) and incubated overnight at 37°C and 180 rpm. Fluorescence measurement was made at two time points: *T*_0_: 24 h and *T*_1_: 48 h post-inoculation. For time point *T*_1_, a 1:10 dilution of the T0 culture was made in M9TG media supplemented with chloramphenicol in another 96-well, 2 ml Master Block (Greiner Bio-One, 780271), and left O/N with shaking of 180 rpm until completion of 48 h.

### Fluorescence measurement

A 1:10 dilution of the overnight culture was prepared in sterile PBS (NaCl 137 mM, KCl 2.7 mM, Na_2_HPO_4_ 10 mM, KH_2_PO_4_ 1.8 mM) in a black 96-well µClear plate (CellStar/Greiner Bio-One, 655090) and read on a BioTek Synergy H1 Microplate Reader. The instrument was set to 25°C, and each plate was linearly shaken for 30 s at 425 cpm (3 mm) before measurement. Optical density at 600 nm was recorded per well. GFP and RFP fluorescence were read with excitation/emission pairs 488/512 nm (GFP) and 532/588 nm (RFP), using 80 and 127 gains, respectively.

Fluorescence values were corrected for OD₆₀₀ and then normalized to the corrected fluorescence of the catalytic-dead control dLbCas12a (E925A/D1180A) (Supplementary Fig. S2). To avoid pseudo replication, the technical replicates within each biological replicate were normalized to that dataset’s own OD₆₀₀ and GFP/RFP values for the dead variant. This approach was applied to all biological replicates at both timepoints. After the double normalization, the replicates were averaged and plotted as three different data points.

### Protein expression and purification for cleavage assay

His₆-tagged LbCas12a variants were expressed in *E. coli* Rosetta (DE3) following a protocol adapted from [44]. Cultures were grown in LB at 37°C to an OD₆₀₀ of ∼0.6, induced with 0.5 mM IPTG, and incubated overnight at 18°C. Cells were harvested, resuspended in lysis buffer (500 mM NaCl, 20 mM Tris–HCl pH 8.0, and 10 mM imidazole), and lysed by sonication on ice. Clarified lysates were applied to Ni-NTA agarose resin pre-equilibrated in the same lysis buffer. The resin was washed with buffer containing 20–30 mM imidazole (500 mM NaCl, 20 mM Tris–HCl pH 8.0, and 20 mM imidazole), and proteins were eluted with 250–300 mM imidazole (500 mM NaCl, 20 mM Tris–HCl pH 8.0, and 250 mM imidazole). Eluted fractions were concentrated and buffer-exchanged into storage buffer (500 mM KCl, 20 mM HEPES pH 8.0, 1 mM DTT added fresh), aliquoted, flash-frozen in liquid nitrogen, and stored at −80°C.

### DNA cleavage assay

*In vitro* nicking assays were performed in 20 µl reactions using either supercoiled plasmid DNA or Cy3/Cy5-labeled linear DNA duplexes as substrates. Reactions followed a protocol adapted from [44] and were assembled in cleavage buffer (20 mM HEPES pH 7.5, 100 mM NaCl, 5 mM MgCl_2_, and 1 mM dithiothreitol (DTT)). The EcoRI and Nb.BbvCI restriction-enzyme controls were performed in the manufacturer’s CutSmart buffer, whereas the LbCas12a–Lid2 reactions used the cleavage buffer described above, with the MgCl_2_ concentration adjusted as indicated for the titration. Cas12a ribonucleoprotein (RNP) complexes were formed by pre-incubating 200 nM crRNA with 100 nM LbCas12a at room temperature for 10 min. Following RNP assembly, 200 ng (approximately 113 fmol; 5.6 nM final concentration) of supercoiled pUC19 (2686 bp) or 2 ng (approximately 51 fmol; 2.5 nM final concentration) of the 60 bp Cy3/Cy5-labeled duplex was added. Reactions were incubated at 37°C for 30 min (for 1 h in case of the supercoiled-plasmid variant comparison) and quenched with 6× DNA loading dye. Supercoiled substrates were resolved by agarose gel electrophoresis and visualized on a standard gel documentation system. Fluorescently labeled duplexes were resolved by denaturing polyacrylamide gel electrophoresis and imaged on a Typhoon scanner (Cytiva). All plasmid constructs and selected cleavage products were verified by Sanger sequencing (Macrogen Europe).

### Protein expression and purification for cryo-EM analysis

The recombinant LbCas12a–Lid2 protein was expressed and purified following a previously described protocol [45]. Briefly, N-terminally His₆-tagged LbCas12a–Lid2 was expressed in *E. coli* Rosetta2 (DE3). The expression strain was cultured at 37°C in 1 l of LB medium supplemented with 20 mg/l kanamycin until the OD₆₀₀ reached 0.8. Protein expression was induced by the addition of 0.1 mM IPTG (Nacalai Tesque), followed by incubation at 20°C for 18 h. Cells were harvested by centrifugation at 5000 × *g* for 10 min, resuspended in lysis buffer containing 20 mM HEPES-NaOH (pH 7.6), 20 mM imidazole, 1 M NaCl, and lysed by sonication. The lysate was clarified by centrifugation at 10 000 × *g* for 30 min. The resulting supernatant was incubated with 0.3 ml of Ni-NTA Superflow resin (QIAGEN) pre-equilibrated with the lysis buffer and loaded onto a Poly-Prep Column (Bio-Rad). Bound proteins were eluted using a buffer containing 20 mM HEPES-NaOH (pH 7.6), 300 mM imidazole, and 300 mM NaCl. The eluate was further purified using a HiTrap Heparin HP column (GE Healthcare) equilibrated with 20 mM HEPES-NaOH (pH 7.6) and 300 mM NaCl, and eluted with a linear NaCl gradient (0.3–2 M). The purified protein was stored at −80°C until use.

The crRNA was synthesized *in vitro* using T7 RNA polymerase and a partially double-stranded DNA template. Following transcription, crRNA was purified by 8% denaturing urea–PAGE, extracted from gel slices using Tris–borate–EDTA buffer (Takara), and recovered by ethanol precipitation. The resulting RNA pellet was dissolved in nuclease-free water and stored at −80°C.

### Cryo-EM sample preparation and data collection

The LbCas12a–Lid2–crRNA–target DNA complex was reconstituted by mixing the purified LbCas12a–Lid2, the 40-nt crRNA, the 39-nt target DNA, and the 39-nt non-target DNA at a molar ratio of 1:1.2:1.2:1.2 at room temperature for 10 min. The reconstituted ternary complex was purified by size-exclusion chromatography on a Superdex 200 Increase 10/300 column (GE Healthcare), equilibrated with SEC buffer composed of 20 mM HEPES-KOH (pH 7.4), 100 mM NaCl, 10 mM MgCl_2_, and 1 mM DTT. The purified complex solution were concentrated until the *A*_260_ reaches 4.0, and then applied to amylamine-treated Au 300-mesh R1.2/1.3 grids (Quantifoil) that were glow-discharged in a Vitrobot Mark IV (FEI) at 4°C, with a waiting time of 10 s and a blotting time of 4 s under 100% humidity conditions [45]. The grids were plunge-frozen in liquid ethane and cooled to the temperature of liquid nitrogen.

Micrographs for all datasets were collected with a Titan Krios G3i microscope (Thermo Fisher Scientific) running at 300 kV and equipped with a Gatan Quantum-LS Energy Filter (GIF) and a Gatan K3 Summit direct electron detector in the electron counting mode (The University of Tokyo, Japan). Datasets were collected with a total dose of approximately 50 electrons per Å^2^ per 48 frames by the standard mode, using the EPU software (Thermo Fisher Scientific). The dose-fractionated movies were subjected to beam-induced motion correction and dose weighting using Patch Motion Correction, and the contrast transfer function (CTF) parameters were estimated using Patch-based CTF estimation in cryoSPARC v4.4.0 [46].

### Cryo-EM data processing

Data were processed with the cryoSPARC v4.4.0 software platform [46]. From the 5403 motion-corrected and dose-weighted micrographs, 7 190 848 particles were automatically picked using Template Picker. The particles were subjected to heterogeneous refinement using maps derived from *ab initio* reconstruction as templates, resulting in the reconstruction of five distinct classes. Two of these classes were subjected to 2D classification and additional rounds of heterogeneous refinement, resulting in the 748 455 selected particles. The particles were then subjected to non-uniform refinement, CTF refinement, and reference-based motion correction. Non-uniform refinement after postprocessing yielded a map with an ambiguous density around the RuvC and TNB domains. 3D classification without alignment using a focused mask around the RuvC and TNB domains improved the density around the RuvC and TNB domains, and the non-uniform refinement after 3D classification yielded a map at an overall resolution of 2.54 Å, according to the Fourier shell correlation (FSC) criterion of 0.143 (Supplementary Fig. S6). The local resolution was estimated by BlocRes implemented in cryoSPARC.

### Model building and validation

The models were built using the crystal structure of LbCas12a (PDB: 5XUU) as reference [15], followed by manual model building with Coot [47, 48]. The model was refined using Servalcat against unsharpened half maps with external restraints prepared by ProSMART and LIBG [49–51]. The model was validated using MolProbity. Molecular graphics figures were prepared with UCSF ChimeraX-1.7.1 [52, 53]. Data collection, processing, model refinement, and validation statistics are summarized in Supplementary Table S4.

## Results

### *In vivo* nicking assay

To distinguish dsDNA-targeting nucleases with SSB (nickase) activity from those with DSB activity, we established an *in vivo* reporter assay in *E. coli* using two fluorescent plasmids, pGFP and pRFP (Fig. 1A). TheI pGFP plasmid carries a single protospacer, whereas pRFP contains two adjacent protospacers. Both reporter plasmids also carry their own crRNA cassette, expressed from the J23119 promoter (Supplementary Fig. S9). We assumed that under non-selective conditions in the absence of antibiotics, paired nicking of pRFP results in a DSB that will cause plasmid loss, whereas a single nick in pGFP is tolerated because SSBs are repaired relatively quickly in *E. coli*; hence, they do not compromise plasmid stability *in vivo*. This design generates three readily distinguishable phenotypes: white colonies for nucleases that induce DSBs (GFP⁻/RFP⁻), orange colonies for catalytically inactive variants (GFP⁺/RFP⁺), and green colonies for nickase variants (GFP⁺/RFP⁻). A variant is scored as a nickase when the GFP signal is retained but the RFP signal is lost (the GFP⁺/RFP⁻ criterion).

**Figure 1.**
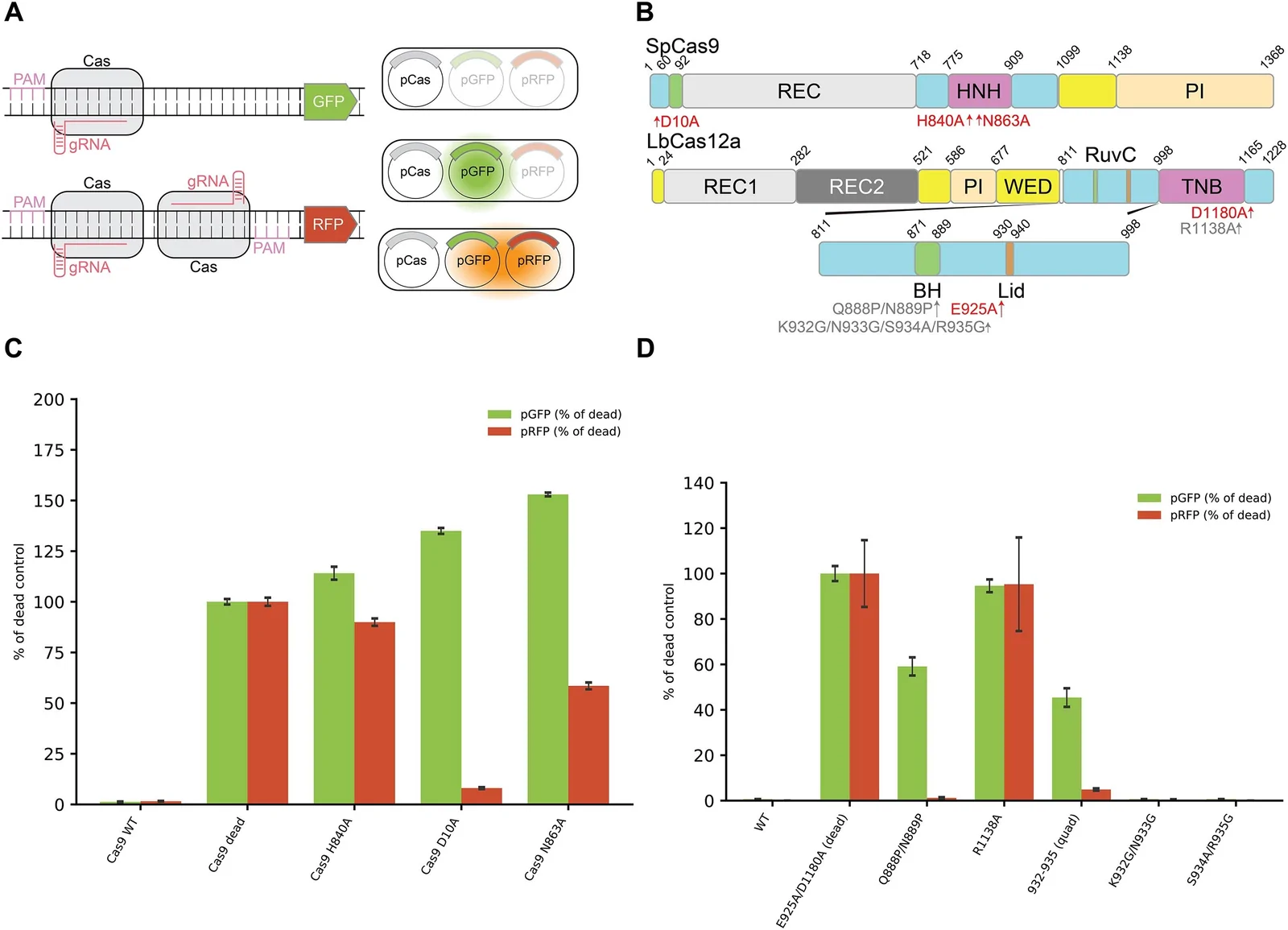
*In vivo* screening assay for Cas9 and Cas12 nickases. (**A**) Schematic of the *in vivo* screening assay in *E. coli*. The assay distinguishes three phenotypes based on plasmid survival: wild-type (DSB generation leads to plasmid loss; white colonies), nicking (SSB allows plasmid survival; green colonies, GFP+/RFP−), or dead variants (no cleavage; orange colonies, GFP+/RFP+). (**B**) Schematic representation of SpCas9 and LbCas12a proteins (not to scale) highlighting the active site residues (red) and the positions of nickase mutations utilized in this study (black). (**C**) Fluorescent phenotype quantification (GFP, green; RFP, red) comparing wild-type (WT) SpCas9 with dead Cas9 (D10A/H840A), TS-nickase (D10A), and two NTS-nickases (H840A and N863A). (**D**) Fluorescent phenotype quantification of LbCas12a variants, comparing WT with dead Cas12a (E925A/D1180A), two previously reported nickase-like variants (Q888P/N889P; R1138A), and the structure-guided Lid mutants designed in this study.

Unlike previously described bacterial and mammalian CRISPR–Cas reporter systems, the selected target sequences on pGFP (PS1) and pRFP (PS1 and PS2) are located in non-coding sequences of the reporter plasmids, not in the genes encoding GFP or RFP themselves. Consequently, Cas12a-induced cleavage and repair in the target window leads to plasmid loss rather than an in-frame indel in the reporter gene, and the GFP or RFP signal is not retained after a DSB. This avoids misclassifying true DSB variants as GFP-positive, which can occur when the target sequence would be located in the coding sequence.

To validate the *E. coli* reporter system, we initially compared several previously characterized SpCas9 variants [7, 13]: wild-type, dead (D10A/H840A), and three nickases (disrupted RuvC activity (D10A), disrupted/attenuated HNH activity (N863A and H840A). Consistent with prior reports, mutants D10A produced a robust nickase phenotype [7, 10, 54, 55]. In contrast, intermediate levels RFP fluorescence was still detectable in case of the N863A and the H840A variants, probably reflecting imperfect paired nicking [55] (Fig. 1B and C). Overall, however, these results confirm that the developed GFP/RFP dual-reporter in *E. coli* provides a reliable *in vivo* system for nuclease variants, that allows for high-throughput screening (colonies on agar plates) and selection (FACS) of libraries of nuclease variants.

We next applied the assay to wild-type LbCas12a and to two previously described LbCas12a nickase variants: a double mutant (Q888P/N889P) reported to affect the Bridge Helix domain [40], and a single mutant (R1138A) identified by systematic mutagenesis study of conserved polar residues in the TNB domain (previously referred to as Nuc domain) [15]. In addition, we decided to analyze variants with substitutions of amino acids of the Lid structure of the RuvC domain, because a recent study indicated that this structure is involved in regulating access of the DNA strands to the active site [37]. The design of two LbCas12a double mutants (K932G/N933G and S934A/R935G) was inspired by a recent study on the Lid of FnCas12a in which both variants were reported to have nicking activity [41, 56]. Tests of the two double mutants in our *in vivo* screening system, however, did reveal that under these conditions the activity of these variants was indistinguishable from the wild-type enzyme (Fig. 2B). Interestingly, a combination of the two variants resulting in a quadruple mutant (K932G/N933G/S934A/R935G) did result in an LbCas12a variant with a nickase phenotype (GFP+/RFP−) (Fig. 2B).

**Figure 2.**
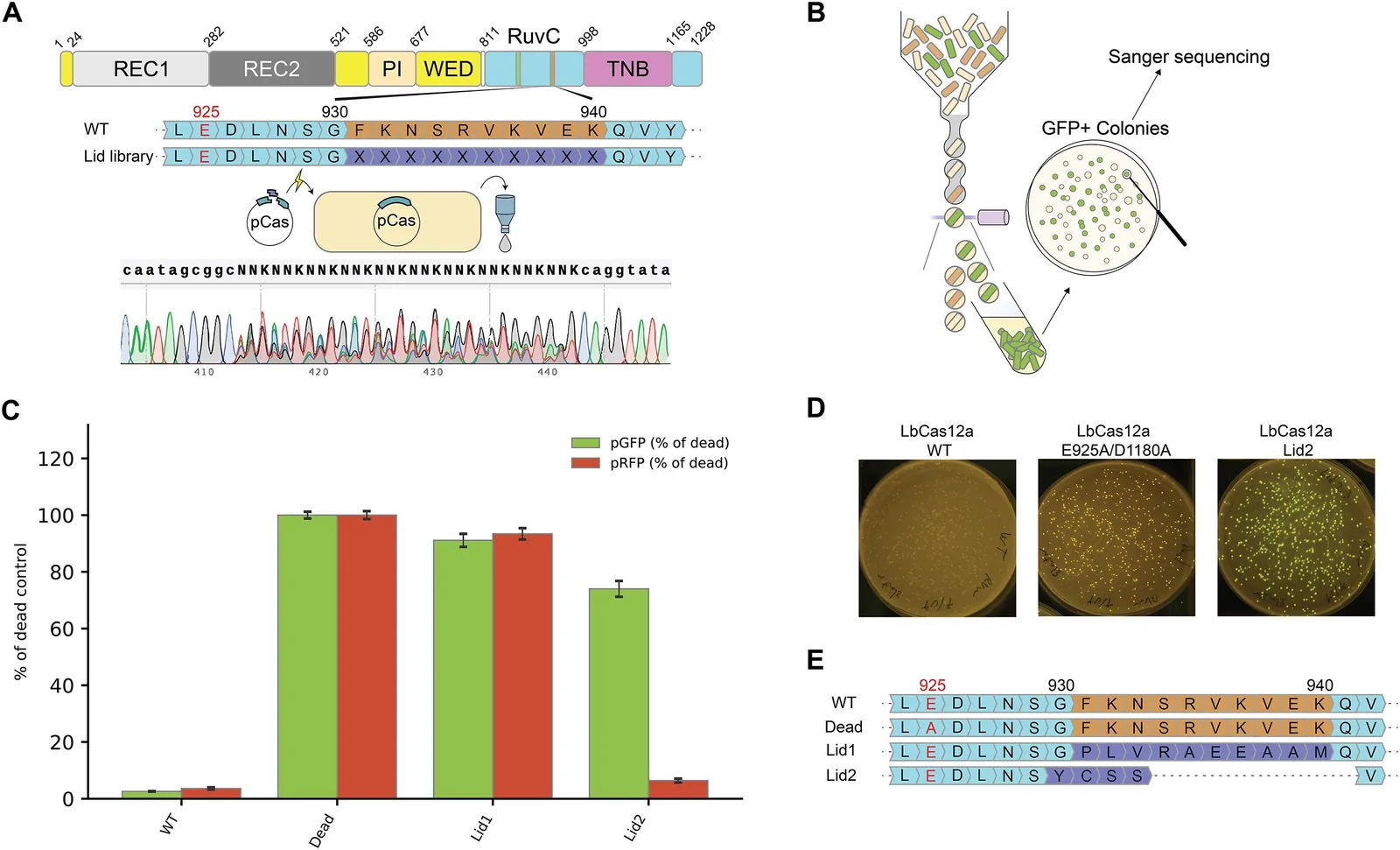
Screening the LbCas12a Lid domain library for nickases. (**A**) Design of the Lid saturation mutagenesis library targeting residues 931–940 and confirmation of the mutagenesis window by Sanger sequencing. (**B**) Workflow of the FACS-based enrichment. The library was subjected to two rounds of sorting for GFP + cells to enrich robust nickase candidates. (**C**) Validation of isolated clones. Unlike the initially analyzed Lid1 variant (column 3; in next section, this variant is referred to as Lid1.10), the Lid2 variant (column 4) reproducibly recapitulates the nickase phenotype (high GFP, low RFP) compared to WT (low fluorescence) and dead (high GFP and RFP fluorescence) controls. Error bars represent mean ± SD (*n* = 3). (**D**) Representative agar plates containing LbCas12a WT (white), dLbCas12a (E925A/D1180A) orange), and LbCas12a–Lid2 (green), displaying the DSB, dead, and nickase phenotypes, respectively. (**E**) Amino acid sequence alignment showing the altered sequences of the Lid1 variant (designed NNK-10, substitution of 10 amino acids) and Lid2 variant (substitution of 12 aa G930Y–Q941 by 4 aa YCSS) compared to WT and Dead (E925A/D1180A) sequences.

Subsequent structural modeling of the wild-type LbCas12a (AlphaFold2 and AlphaFold3; data not shown) supported an important role of the Lid domain by predicting specific intra-molecular contacts by some Lid residues (positions 931–940). For example, the side-chain oxygen of N933 (Lid) was predicted to interact with the phosphate backbone of the crRNA of the guide-target heteroduplex at position 9, while intra-protein contacts were predicted between K932 (Lid) and E947 (RuvC), between R935 (Lid) and E835 (RuvC). In addition, earlier structural studies revealed interactions of F931 (Lid) with the NTS [34], and of E939 (Lid) with R883 and R887 (Bridge Helix) [34]. Consistent with this, analogous Lid residues (K1013, R1014, R1016, and K1018) in FnCas12a anchor the PAM-distal part of the heteroduplex [38], supporting a conserved role for Lid-mediated nucleic-acid and intradomain contacts across Cas12a orthologs. An alignment of the Lid region across well-characterized Cas12a orthologs (LbCas12a, FnCas12a, and AsCas12a; Supplementary Fig. S8) shows that the basic residues K932 and R935 are very well conserved, whereas N933 and S934 are more variable, suggesting that the former two Lid residues are most critical for catalytic regulation in the Cas12a family. Together, these interactions likely stabilize the structure and the position of the α-helical Lid, and they may play an important role in the eventual translocation of the TS toward the RuvC active site.

The Q888P/N889P double mutant may indicate an important role of the Bridge Helix in cleavage of the second strand. In addition, the R1138A mutant and the new quadruple mutant (K932G/N933G/S934A/R935G) may play a role in coordinating NTS and TS cleavage for the TNB domain as well as for the α-helical Lid of the RuvC domain, respectively. These findings are consistent with structural and biochemical evidence that Cas12a cleaves the two DNA strands sequentially and in a coordinated manner [6, 17, 35]. Beyond the Bridge Helix, recent work has emphasized the importance of the Lid domain in orchestrating this two-step mechanism [35]. In conjunction with these findings, our *in vivo* reporter analysis strengthens the notion that the Lid is a crucial regulatory element determining Cas12a nuclease activity. This motivated us to focus on the Lid region by performing a targeted mutagenesis screen of this sub-domain with the goal to identify variants with robust nickase activity.

Using the initial *in vivo* screen, potential variants with the desired phenotype are selected for subsequent *in vitro* comparison. The rationale is that single nicks can be repaired (hence plasmids will survive), but that adjacent double nicking of opposite strands generate DSBs that will generally result in plasmid loss. In addition, also in the absence of plasmid linearization (DSBs) there will be spontaneous plasmid loss (“curing”) due to lacking selective pressure (in the absence of antibiotics). In case of a lost reporter plasmid, a color change of the corresponding colony depends on the half-life of the reporter, GFP in the case of the nickase screen used in this study. In practice, active plasmid attack can be distinguished from spontaneous curing within a window of 24–48 h, under the cultivation conditions described in the Methods section.

### Identification of LbCas12a variants with *in vivo* nickase activity

We next applied the dual-fluorescent reporter system to a saturation mutagenesis library of the LbCas12a Lid domain. Based on the preliminary screen that revealed nickase-like phenotypes, we focused on a stretch of ten Lid residues (F931–K940) to examine their potential role in coordinating sequential cleavage. To comprehensively interrogate this region, we introduced NNK codons (N = A/C/G/T, K = G/T) across all consecutive positions, generating a saturation mutagenesis library spanning this 10 amino acid segment (Fig. 2A).

The Lid1 NNK-10 saturation library was transformed into the *E. coli* dual-reporter strain carrying pGFP and pRFP. After incubation in liquid culture for approximately 18 h, the obtained cells were washed in a PBS buffer, after which the GFP⁺ cells were recovered from the population by FACS. Sorted populations were plated on LB agar supplemented with chloramphenicol to maintain the Cas variant plasmid, but without antibiotics for the GFP and RFP reporters, thereby allowing detection of reporter plasmid loss driven specifically by nickase or DSB activity. Twelve GFP⁺ colonies were selected for Sanger sequencing. In the initial selection, only a single designed NNK-10 mutant (Lid1) was obtained with 10 amino acid substitutions at positions 931–940. Surprisingly, the predominant variant (8/12 isolates), hereafter designated Lid2 (Fig. 2B), had a drastically restructured Lid domain with four non-synonymous substitutions (G930Y, F931C, K932S, and N933S) and an in-frame deletion of eight amino acids (S934–Q941) (Fig. 2E). This genotype most likely originated from a library construction artifact (Supplementary Fig. S1). The remaining three isolates were attributed to cloning artifacts (major deletions) of the NNK library and were not pursued further. All twelve clones were re-cloned and re-analyzed in the dual-reporter strain. Four isolates from the original screen (including Lid1) did not survive re-cloning (Fig. 2C), most likely because of RFP loss due to instability of the RFP reporter without antibiotic selection. Interestingly, the Lid2 clone reproducibly was GFP⁺/RFP⁻ (Fig. 2C and D), corresponding to a robust nickase phenotype.

To dissect which Lid alterations (sequence and/or size) are sufficient for nickase activity, four different strategies were designed: (i) NNK-10 library of the wild-type Lid (Lid1 series), (ii) systematic, step-wise trimming of the wild-type sequence both from C-terminal end (Lid1Δ-C series) and from N-terminal end (Lid1Δ-N series), (iii) NNK-4 library of the YCSS Lid (Lid2 series), and (iv) systematic, step-wise trimming of the YCSS Lid from the C-terminal end (Lid2Δ#-C). In the following section, the results of each of these strategies are described.

We first set out to perform a more extensive analysis of the initially designed Lid1 series. As described above, the NNK-10 saturation library was generated to randomize the core Lid residues at positions 931–940 (Fig. 2E). Nine library members with distinct patterns of Lid substitutions were re-cloned individually and assayed in the dual-fluorescent *E. coli* reporter strain alongside wild-type LbCas12a and the catalytic-E925A/D1180A control (Fig. 3A). Seven variants (Lid1.1, Lid1.2, Lid1.5, Lid1.6, Lid1.7, Lid1.8, and Lid1.9) reproduced the GFP+/RFP− nickase phenotype with normalized RFP signal between approximately 18 and 34 units relative to the dead control set to 100 (Fig. 3A). The remaining two variants (Lid1.3 and Lid1.4) displayed mixed phenotypes that did not satisfy the GFP+/RFP− criterion. One of the best performing nickases concerned Lid1.5, in which F931, K932, N933, and E939 are conserved, but S934 and R935 are substituted (Fig. 3A). As indicated above (Fig. 1D), three more Lid1-like variants are generated, i.e. the two double mutants with a wild-type phenotype, and the combined quadruple mutant (K932G/N933G/S934A/R935G) with a nickase phenotype. These results show that our screening platform allows for recovering a wide range of Lid substitution variants with nickase activity, supporting the conclusion that the Lid sub-domain tolerates a range of substitution patterns compatible with the uncoupling of NTS and TS cleavage. The Lid sequences and nickase phenotypes of all engineered Lid variants assessed in this study, grouped by engineering series, are provided in Supplementary Table S5.

**Figure 3.**
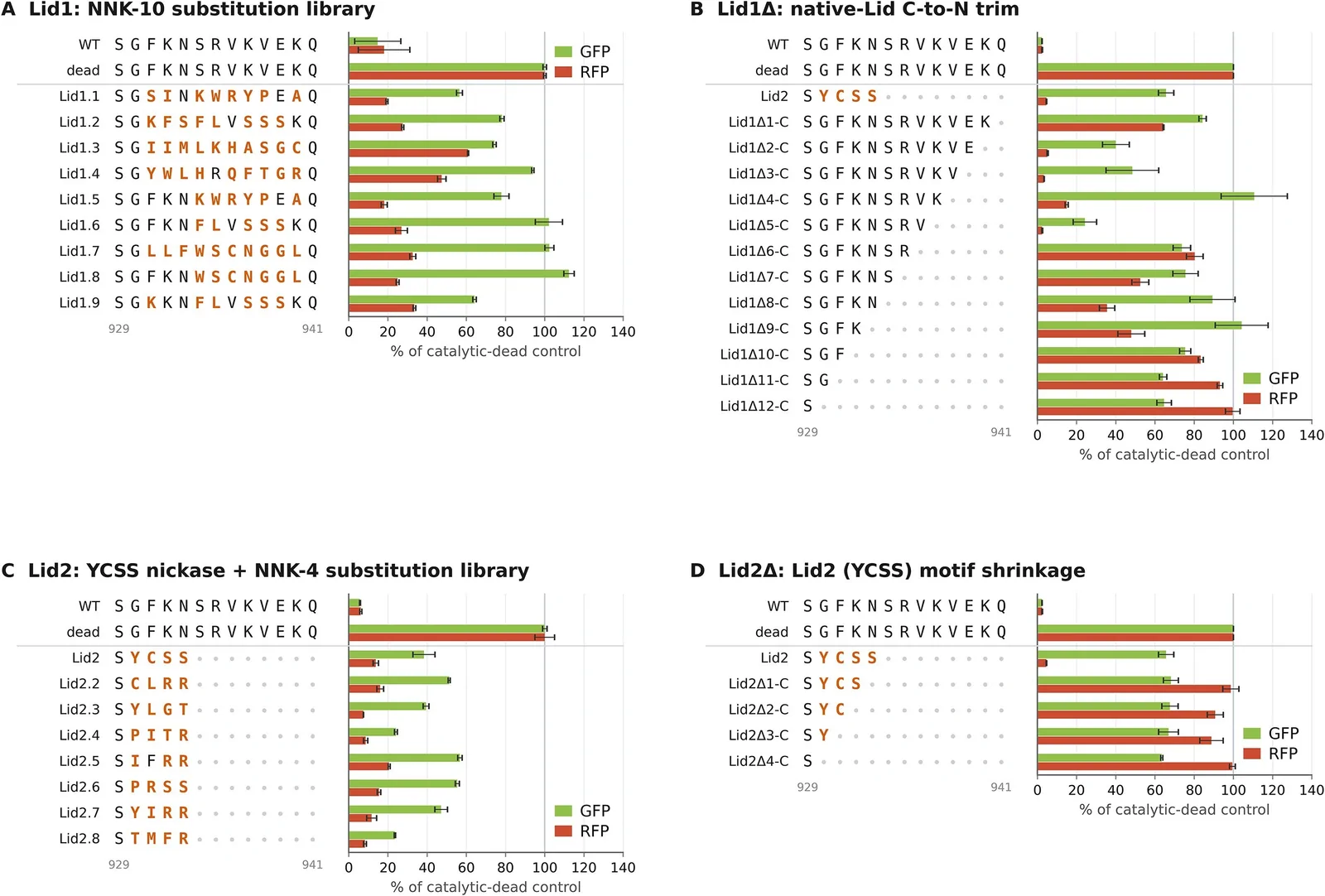
Sequence and length variation of the Lid defines the residues required for nickase activity. Engineered LbCas12a Lid variants, grouped into four families, were screened in the dual-fluorescent reporter assay against wild-type LbCas12a and the catalytic-dead E925A/D1180A control; each panel shows GFP and RFP reporter signal from a single overnight culture (Supplementary Fig. S3 shows a second time point after re-inoculation and overnight incubation, with the C-to-N and N-to-C truncation series compared at both time points), as a percentage of the catalytic-dead control (mean and SEM of three biological replicates) above the matched 929 to 941 Lid amino-acid alignment. (**A**) Lid1: the NNK-10 substitution library (Lid1.1–Lid1.9). (**B**) Lid1Δ: progressive C-to-N truncation of the native Lid ending at the fully deleted Lid (Lid1Δ1-C to Lid1Δ12-C); the N-to-C truncation series (Lid1Δ1-N to Lid1Δ12-N) is provided in Supplementary Fig. S3E and F. (**C**) Lid2: the YCSS nickase together with the NNK-4 substitution library at positions 930 to 933 on the Lid2 scaffold (Lid2.2–Lid2.8). (**D**) Lid2Δ: the Lid2 (YCSS) motif progressively truncated, shown from the intact motif (Lid2) through Lid2Δ1-C to Lid2Δ4-C.

We then executed a systematic Lid-trimming panel of the native-Lid for generating the Lid1Δ series. All variants were screened in the dual-fluorescent reporter system, alongside wild-type LbCas12a, the catalytic-dead E925A/D1180A control, and the original Lid2 (YCSS) mutant. Plate-reader endpoint measurements were taken at 24 and 48 h post-induction. In the Lid1Δ series, we progressively trimmed the native Lid of the wild-type backbone: the C-terminal trim (Lid1Δ1-C to Lid1Δ12-C; 24 h) (Fig. 3B) and the N-terminal trim (Lid1Δ1-N to Lid1Δ12-N; 24 h) (Supplementary Fig. S3); for the results of the 48 h post-induction experiments, see Supplementary Fig. S3. The phenotypes of the trimmed variants spanned the full nickase-to-inactive range. At least a dozen of variants were obtained with potential nickase activity. Notably, a Lid containing the N-terminal eight wild-type residues (Δ938–941; Lid1Δ4-C) resulted in a nicking activity comparable to that observed for Lid2 (Fig. 3B). Interestingly, the best performing variant from the N-terminal trimming also is the result of a 4-aa deletion, consisting of the C-terminal eight amino acids (Δ930–933; Lid1Δ4-N). Still, with the current data set, and in the absence of additional structural insights, it is not possible to comprehend why certain Lid variants have a nickase phenotype (reasonable NTS cleavage, poor TS cleavage), whereas other variants show relatively low overall activity (poor cleavage of both NTS and TS). However, the observation that the wild type activity of generating double-strand DNA breaks is disrupted in almost all variants with deletions of 1–12 amino acids, confirms the crucial function of the Lid domain in the Cas12a mechanism. In fact, the only exception of a here described deletion variant with an apparent wild-type performance is Lid1Δ1-N, i.e. carrying a deletion of a single amino (G930) that is positioned just outside the here defined Lid core region (931–940).

The Lid2 variant was next taken as a starting point for an NNK-4 library that varied all four positions of the YCSS Lid. Seven additional variants were analyzed (Fig. 3C); all shared a similar profile, with GFP fluorescence at least three-fold higher than RFP fluorescence. As with the parental Lid2, two of these variants, Lid2.3 and Lid2.7, carry a tyrosine at position 930 (Y930), the potential relevance of which is discussed below. Notably, these Lid2 variants and the parental Lid2 all retain a Lid carrying an eight-residue deletion, consistent with the nickase phenotype seen for the length-matched Lid1Δ8-C and Lid1Δ8-N variants. Systematic truncation of the YCSS sequence on the Lid2 scaffold (the Lid2Δ series, Lid2Δ1-C to Lid2Δ4-C) disrupted nickase activity (Fig. 3D).

Many different LbCas12a variants with nickase phenotypes have been generated here by introducing changes in the Lid sequence (Figs 2 and 3). Their combined double-strand-break and nicking activities, scored relative to the catalytic-dead control, are summarized in a phenotype map (Supplementary Fig. S4). It should be kept in mind, however, that the described phenotypes are based on the here obtained GFP and RFP fluorescent data (Figs 2 and 3), and that these results may slightly differ when using another screening approach, and/or when using different guide-target pairs. To allow for in depth molecular analysis, we decided to focus the present study on the original Lid2 clone. The reason for selecting this clone is not only because of its robust nickase phenotype across the *in vitro* and *in vivo* assays, but also because of its distinctive genotype based on a substitution of 12 codons of the Lid core sequence of residues at positions 930–941 (GFKNSRVKVEKQ) by four completely different ones (YCSS). Hence, the essential core Lid has been changed dramatically, both in terms of size and sequence. Compared to previously reported Cas12a nickases, this implies that the Lid2 variant is a completely novel Cas12a nickase variant. Below, an in-depth functional and structural characterization of LbCas12a–Lid2 is described.

### LbCas12a–Lid2 displays an NTS nickase phenotype *in vitro*

To validate the strand-selective nicking activity of the LbCas12a–Lid2 variant, we performed *in vitro* cleavage assays with recombinant protein. Using a negatively supercoiled plasmid substrate, DNA topologies were visualized by agarose gel electrophoresis, where nicked plasmids migrate more slowly than linearized or supercoiled forms (Fig. 4A, lanes 1–3). In comparison with wild-type LbCas12a and three previously described Cas12a variants, Lid2 showed substantial accumulation of the nicked product, confirming a robust nickase phenotype (Fig. 4A, lane 12). The lower overall cleavage activity observed on the linear oligonucleotide substrate (Fig. 4B) compared with the supercoiled plasmid substrate (Fig. 4A) is consistent with the requirement of negative supercoiling for efficient R-loop formation, in agreement with prior reports for both Cas9 and Cas12a effectors [57, 58]. The oligonucleotide assay therefore does not measure absolute activity and remains useful for resolving NTS versus TS cleavage at single-strand resolution, while the plasmid assay is the more physiological readout for absolute rates. The bridge-helix Q888P/N889P variant [40] is shown in Fig. 4A only as a reference to a previously described nickase-like LbCas12a mutant; it was not characterized kinetically in this study. Nicking by Lid2 increased with MgCl_2_ concentration while double-strand cleavage remained minor, consistent with a Mg^2+^ dependent RuvC mechanism (Supplementary Fig. S5B and C).

**Figure 4.**
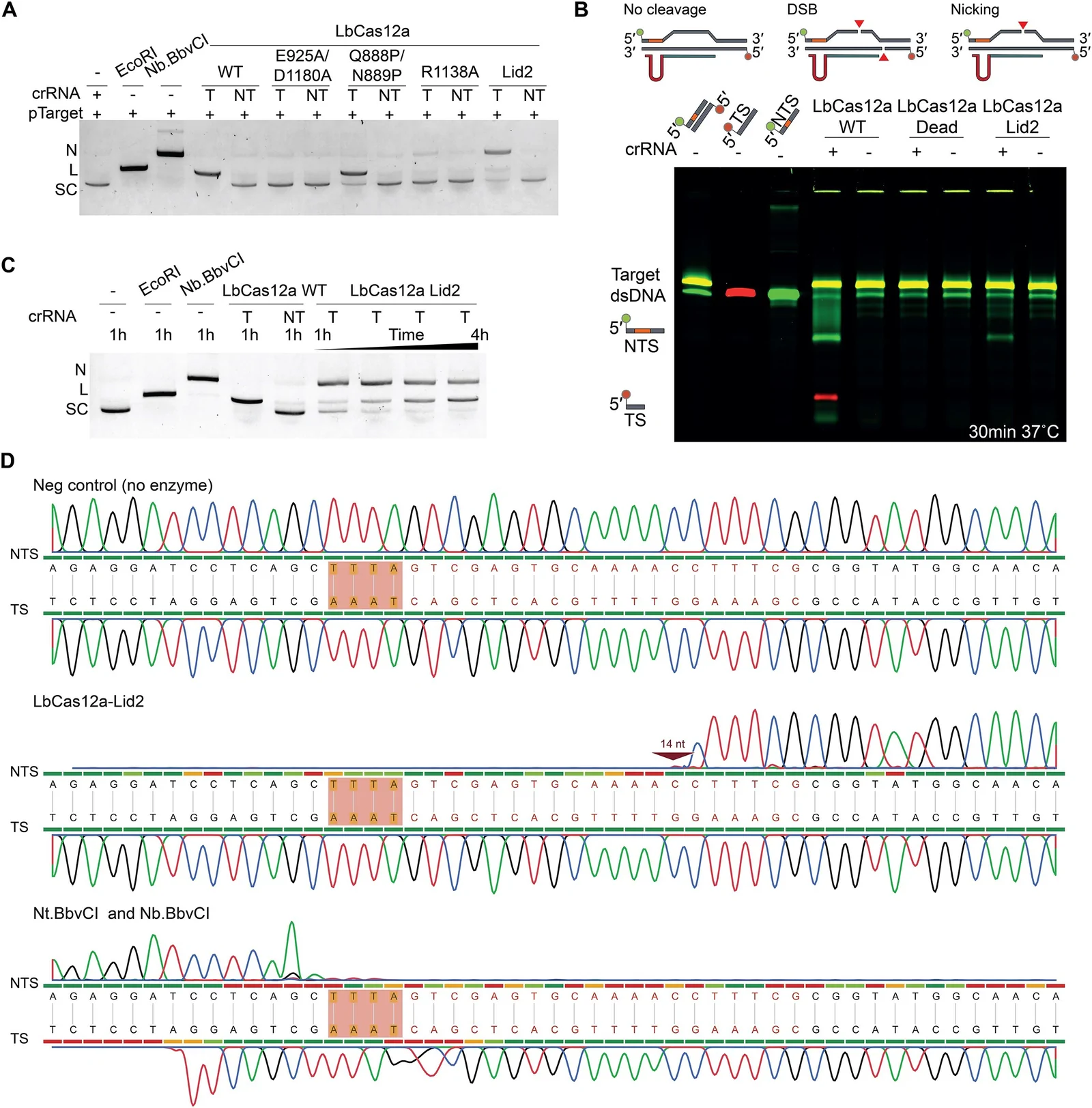
In vitro characterization of LbCas12a–Lid2. (**A**) *In vitro* cleavage assay using a supercoiled plasmid substrate to compare wild-type LbCas12a (WT), catalytically inactive Cas12a (E925A/D1180A), two previously reported nickase-like variants (Q888P/N889P and R1138A), and the Lid2 variant. All reactions were incubated at 37°C for 1 h. The migration patterns indicate Nicked (N), Linear (L), and Supercoiled (SC) plasmid forms. Lanes 1–3 are the topology references (no enzyme, EcoRI-linearized and Nb.BbvCI-nicked plasmid), and each Cas12a variant is assayed with a targeting (T) and a non-targeting (NT) crRNA, the latter serving as the guide-specificity control. (**B**) Denaturing urea–PAGE of a fluorescently labeled linear dsDNA substrate (5′-Cy3 on NTS; 5′-Cy5 on TS). The gel shows that while WT LbCas12a cleaves both strands, the Lid2 variant specifically generates the NTS cleavage product. Each enzyme is assayed with (+) and without (−) crRNA, the latter serving as the guide-dependence control. (**C**) Time-course assay (1–4 h) comparing the cleavage progression of WT and Lid2. WT is shown with a targeting (T) and a non-targeting (NT) crRNA at 1 h, and the Lid2 series is the targeting-crRNA time course. Lid2 displays robust accumulation of the nicked product (N) with only faint linearized product (L) appearing after extended incubation, indicating a severe impairment of TS cleavage activity. (**D**) Sanger sequencing of the nicked plasmid band generated by Lid2 confirms strand specificity. The traces reveal specific cleavage of the NTS, while the TS remains intact. The red arrowhead marks the reproducible cleavage position, 14 nt downstream of the PAM. The colored bar above each base indicates the Sanger base-call quality (Phred) at that position, from green (a clean, high-quality call) to red (a low-quality or mixed call). Per-position base calls and quality for both strands are listed in Supplementary Table S7.

To determine which strand Lid2 cleaves, we performed cleavage assays with a 60 bp dsDNA substrate labeled with a 5′-Cy3 fluorophore on the NTS and a 5′-Cy5 fluorophore on the TS (Fig. 4B). Products resolved on denaturing PAGE revealed that wild-type LbCas12a produced two discrete bands corresponding to cleavage of both strands [9]. In contrast, the Lid2 variant only generated the NTS cleavage product, albeit with lower intensity than wild-type, indicating reduced catalytic activity of the mutant. After 60 min at 37°C, a faint linearized plasmid band was detectable (Fig. 4C; quantified in Supplementary Fig. S5A), indicating that Lid2 can still cleave the TS, albeit relatively inefficient compared to the wild-type LbCas12a. Also, in line with the aforementioned experiment (Fig. 4B), comparison of the 1-h incubations (Fig. 4C) indicates that while Lid2 retains robust nicking activity, its overall single-turnover cleavage rate is moderately lower than that of the wild-type LbCas12a, consistent with the kinetic framework for Cas12a substrate engagement and cleavage reported by Strohkendl *et al*. [59]. The intermediate observed in the Fig. 4C time course is consistent with either a single-nick or a short-gap product; the presented *in vitro* data do not unambiguously distinguish between these two states. Distinguishing a clean nick from a short gap would require end-resolved, strand-specific sequencing-gel analysis, which remains to be determined.

The wild-type LbCas12a generates a staggered DSB via sequential RuvC cleavage of the NTS (typically 18 nt downstream of the PAM), followed by the TS (typically 23 nt downstream of the PAM) [9, 17]. To map the Lid2 nick at nucleotide resolution, we analyzed both strands of the nicked product by Sanger sequencing (Fig. 4D), as was previously used to define wild-type cleavage sites [9, 17]. Sequencing reads of the NTS terminated at position 14 downstream of the PAM, whereas TS reads remained continuous. In agreement with previous analyses of the LbCas12a nickase (R1138A) [15], this confirms that Lid2 specifically nicks the NTS.

Together, these results demonstrate that Lid2 still cleaves the NTS (like the wild-type), after which it rarely progresses to TS cleavage (unlike the wild-type), yielding nicked intermediates that accumulate over time. Next, we set out to reveal the molecular basis of the nickase phenotype.

### Cryo-EM structure

To gain mechanistic insights into the nickase phenotype exhibited by the LbCas12a–Lid2 variant, we determined the cryo-electron microscopy (cryo-EM) structure of the LbCas12a–Lid2 variant in complex with both a 40-nt crRNA (including a 20-nt guide sequence) and its complementary dsDNA target (39-bp), at an overall resolution of 2.5 Å (Fig. 5A–D). Except for the disrupted Lid region, the overall architecture of LbCas12a–Lid2 closely resembles the previously reported crystal structure of wild-type LbCas12a [34], indicating that the introduced mutations in the Lid at position 930–941 (substitution of the GFKNSRVKVEKQ peptide by YCSS) do not affect the overall structure of the complex. The pseudoknot structure of the crRNA scaffold firmly interacts with the WED and the RuvC domains, while the PAM-containing DNA duplex is bound to the groove between the REC1 and PI domains. The guide segment of the crRNA base-pairs with the target DNA strand TS to form a 20-bp guide–target heteroduplex, which is accommodated within the positively charged central channel of the REC1 and REC2 domains. Notably, we observed a density corresponding to a single-stranded DNA (ssDNA) substrate within the RuvC active site of the LbCas12a variant, that is not observed in the crystal structure of the wild-type LbCas12a (Fig. 5C). Nucleotides dA10* to dT17* of the displaced non-target DNA strand (NTS) fit well in the density, strongly suggesting that the present structure represents the NTS-cleaving state of LbCas12a–Lid2 (Fig. 5E). The mutated Lid (G930Y, F931C, K932S, and N933S; collectively referred to as the YCSS motif) adopts a loop conformation that bridges strand β4 and helix α2 within the RNaseH fold of the RuvC domain (Fig. 5F and Supplementary Fig. S7). This YCSS motif, together with the RuvC active site (D832, E925, and D1172), forms a narrow substrate-binding pocket that accommodates the NTS (Fig. 5F). The scissile phosphate between dT14* and dG15* is in a cleavage-competent position in the RuvC active site, with the two magnesium ions are coordinated by D832, E925, and D1172, in agreement with the NTS cleavage position 14-nt downstream from the PAM as observed in Sanger sequencing of the nicked DNA product (Fig. 3B). Additionally, Y930 in the YCSS motif forms a hydrophobic interaction with the ribose moiety of dG15*, facilitating the stable binding of the NTS within the RuvC active site. These structural observations strongly indicate that LbCas12a–Lid2 cleaves the NTS in a manner dependent on two magnesium ions, which is typical for all other Cas12 variants with nuclease activity [60].

**Figure 5.**
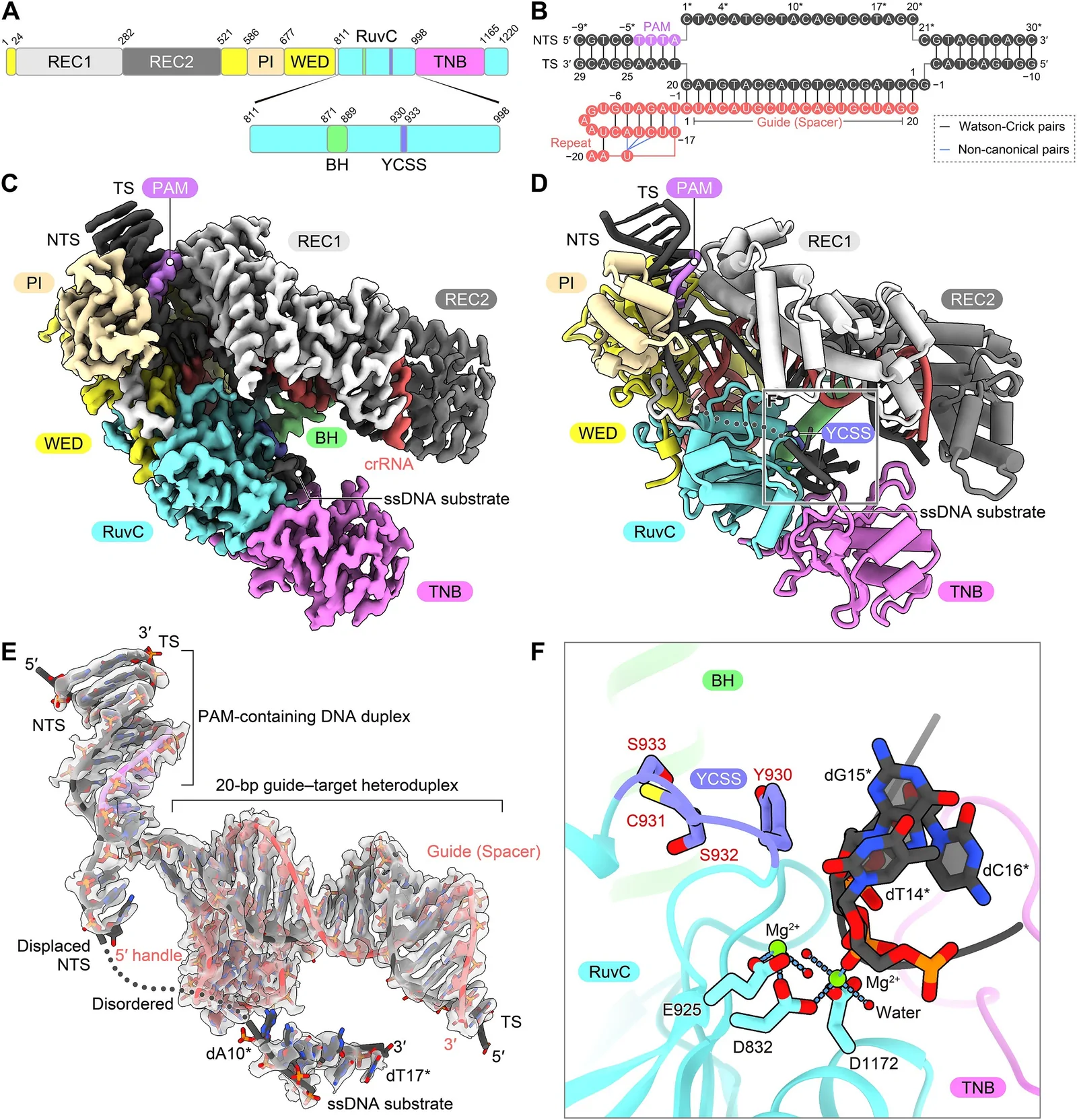
Cryo-EM structure of the LbCas12a–Lid2–crRNA–target DNA ternary complex. (**A**) Domain structure of the LbCas12a–Lid2 mutant. Domains are colored as follows: REC1 (gray), REC2 (dark gray), WED (yellow), RuvC (cyan), and TNB (magenta). (**B**) Schematic of the crRNA and the target DNA interactions. (**C**) Cryo-EM density map (gray surface) of the LbCas12a–Lid2 ternary complex. (**D**) Ribbon model of the complex. The flexible Lid2 loop is visible in the RuvC domain. (**E**) Cryo-EM density map focused on the crRNA and target DNA. The disordered region of the displaced NTS is indicated by the dotted line. (**F**) Close-up view of the RuvC active site and the engineered YCSS motif (residues 930–933). The bound Mg^2+^ ions and water molecules are depicted as light green and red spheres, respectively. **PDB: 9WNE; EMDB: EMD-66115**.

### Structural basis for nickase phenotype

Structural comparison of LbCas12a–Lid2 with the wild-type LbCas12a reveals a molecular basis of the impaired TS cleavage and the resulting nickase phenotype of the LbCas12a–Lid2. In the wild-type LbCas12a structure [34], the Lid adopts an α-helical conformation in which N933 forms a hydrogen bond with G9 in the guide–target heteroduplex (Fig. 6A and B). Additionally, E939 establishes salt bridges with two residues (R883 and R887) of the Bridge Helix (BH), anchoring the α-helical Lid in an open conformation that facilitates accommodation of a ssDNA substrate into the RuvC active site (Fig. 6B). Superimposition of the ssDNA substrate from the LbCas12a–Lid2 structure onto the wild-type LbCas12a further suggests that F931 in the α-helical Lid of the wild-type contributes to substrate stabilization by forming stacking interactions with the nucleobases of the displaced NTS in the RuvC active site (Fig. 6B). In the LbCas12a–Lid2 structure, in contrast, the mutated YCSS motif no longer adopts an α-helical conformation (Fig. 6D), resulting in the loss of interactions with both the guide–target heteroduplex and the Bridge Helix observed in the wild-type LbCas12a. However, the Lid2 variant appears, at least to some extent, to maintain the substrate stabilizing interaction through a hydrophobic interaction between Y930 (in the YCSS motif) and the ribose moiety of the ssDNA substrate (Fig. 6C–F).

**Figure 6.**
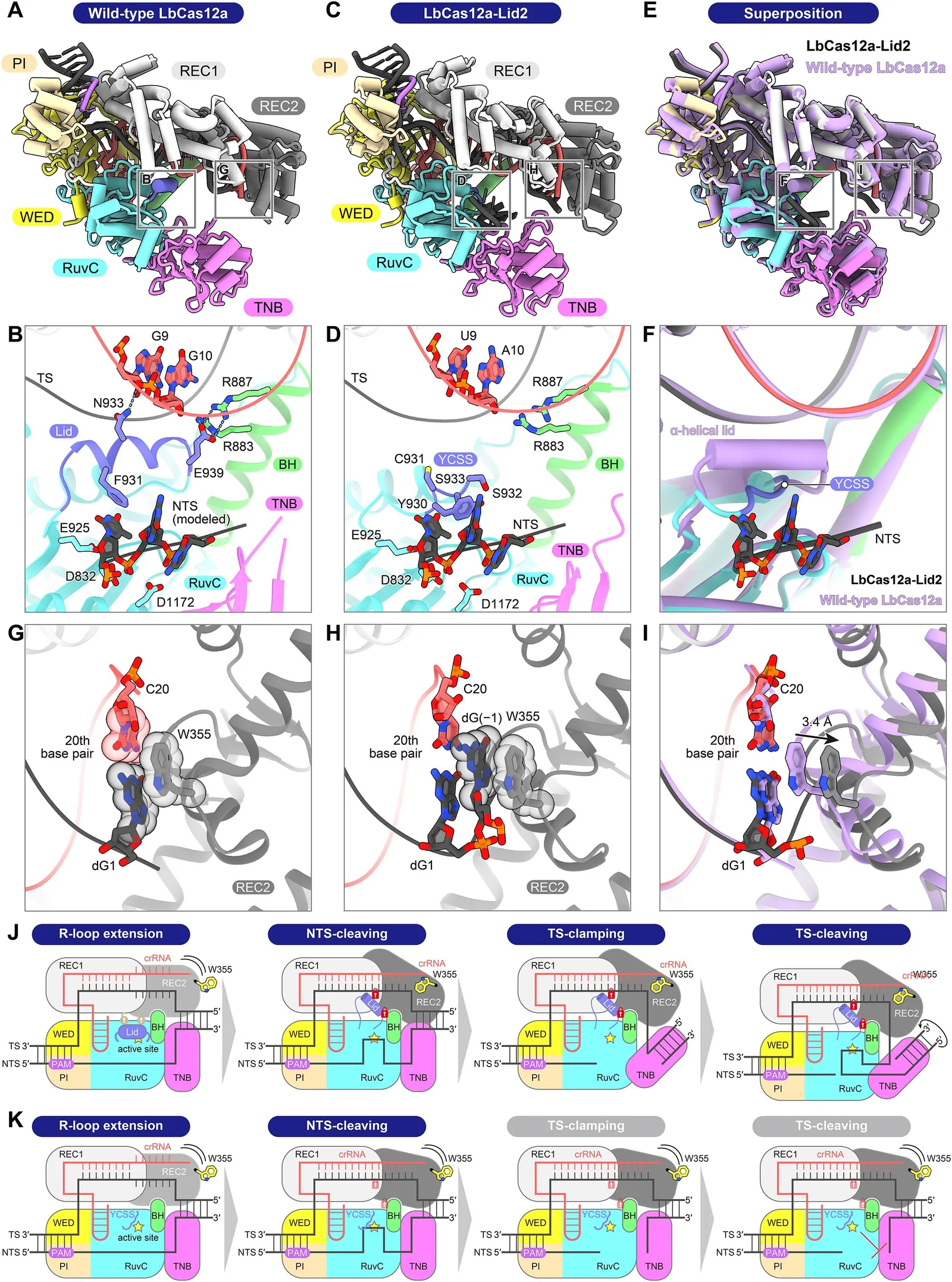
Structural comparison between wild-type LbCas12a and LbCas12a–Lid2. (**A**) Ribbon model of the wild-type LbCas12a–crRNA–target DNA ternary complex (PDB: 5XUU). (**B** and **G**) Close-up views of the wild-type α-helical Lid region (B) and the terminus of the guide–target heteroduplex (G). In panel (B), the substrate NTS was modeled based on the LbCas12a–Lid2 structure. (**C**) Ribbon model of the LbCas12a–Lid2–crRNA–target DNA ternary complex (colored as in Fig. 5). (**D** and **H**) Close-up views of the remodeled YCSS motif (D) and the terminus of the guide–target heteroduplex (H) in the LbCas12a–Lid2 structure. (**E**) Superimposition of the wild-type LbCas12a (pink) and LbCas12a–Lid2 (colored as in C). (**F**) Structural comparison of the Lid region highlighting the loss of the α-helix in the Lid2 mutant. (**I**) Structural comparison around the terminus of the guide–target heteroduplex, showing the 3.4 Å outward shift of W355 in the REC2 domain of the Lid2 mutant. (**J** and **K**) Mechanistic model of LbCas12a wild-type and Lid2-nickase. (J) LbCas12a wild-type: step-wise domain rearrangements allow for sequential cleavage. The α-helical Lid stabilizes the R-loop, facilitating REC2 docking and the subsequent “TS-clamping” conformational change required for TS cleavage. (K) LbCas12a–Lid2 nickase: The remodeled Lid (YCSS loop) stabilizes the NTS for the first nick but fails to stabilize the heteroduplex terminus. This impairs REC2 docking (unlocked state) and prevents the enzyme from adopting the TS-clamping state, thereby restricting activity to NTS nicking only.

In addition, structural comparison further reveals some local conformational differences in the REC2 domain. In the wild-type LbCas12a structure, W355 in the REC2 domain stacks with the 20th base pair of the heteroduplex (Fig. 6G). Interestingly, however, in the LbCas12a–Lid2 structure, W355 undergoes a 3.4-Å outward movement from the terminus of the heteroduplex, potentially making room for accommodating an additional base (Fig. 6H and I). Indeed, we observed a density between dG1 and W355, into which the dG(−1) is well fitted, although it could not form an additional base pair because a crRNA with a 20-nt guide was used during complex reconstruction. This suggests that LbCas12a–Lid2 fails to fully dock the REC2 domain onto the 20th base pair of the heteroduplex, likely due to the loss of stabilizing interactions between the disrupted Lid and the guide–target heteroduplex. Consistent with this structural observation, recent studies suggest that interactions between the α-helical Lid and the guide–target heteroduplex play a pivotal role in docking of the REC2 domain onto the terminus of the heteroduplex, whose conformational change is critical for subsequent TNB domain rearrangement and the eventual TS loading into the active site [17, 35, 38, 59, 61]. Collectively, these structural observations demonstrate that the absence of the α-helical Lid interactions in LbCas12a–Lid2 does disturb the allosteric regulation among the Lid, REC2, and TNB domains that is required for TS loading into the active site, thereby explaining its nickase phenotype: impaired TS cleavage activity while retaining robust NTS cleavage.

## Discussion

CRISPR-associated Cas12a nucleases cleave double-stranded DNA by using a single RuvC catalytic site [17]. Details on the order and regulation of sequential NTS and TS cleavage have gradually been clarified over the past decade [60]. In the present study, we aimed at developing a Cas12a nickase, for fundamental reasons to reveal some elusive mechanistic details, as well as for applied reasons to contribute to expanding the set of Cas12a-based genome editing applications. Based on previously reported Cas12a variants with nickase-like phenotypes, the Lid sub-domain of RuvC was selected as a target. A semi-rational mutagenesis approach was developed in which 10 amino acids of the Lid domain were randomly substituted, and the resulting library was analyzed using a novel *in vivo E. coli* screening system based on GFP and RFP reporters, that allowed for high-throughput screening (on agar plates) and selection (by FACS). Unexpectedly, the generated library not only did, as designed, contain Cas12a variants with Lid domains with up to 10 substitutions (Lid1) but also included a serendipitous substitution of 12 amino acids (including the Lid core) by a completely different four amino acid peptide (Lid2). This discovery highlights the power of the developed unbiased screening system to identify semi-rationally designed (Lid1) as well as non-intuitive (Lid2) structural solutions. More specifically, this has allowed for obtaining a nickase variant in which the ordered α-helical Lid structure is replaced by a flexible loop, a solution that rational design would most likely have overlooked. While the *in vivo* screening system described here is robust, its accuracy is not perfect. Variation in plasmid and reporter-protein concentration, together with differences in nicking efficiency across guide/target combinations, constrain its resolution. The system is therefore best regarded as a qualitative screen whether or not a certain variant acts as a potential nickase, rather than a quantitative one. Still, to compare the different phenotypes, the GFP/RFP fluorescent data have been used to compare the nickase phenotypes of the obtained LbCas12a variants. Some interesting observations are that the Lid2 variant appears to have a robust nickase phenotype, just as the Cas9 nickase control (D10A) and two other LbCas12a variants with Lid deletions (e.g. Lid1Δ3-N, Lid1Δ4-C) as well as the quadruple mutant K932G/N933G/S934A/R935G. Another variant with a convincing nickase phenotype concerns the previously reported LbCas12a mutant with a disrupted Bridge Helix (Q888P/N889P) [40]. In contrast, no nickase phenotype was obtained in our analysis of the previously reported LbCas12a variant with a mutation in the TNB domain (R1138A) [15], most likely caused by variability due to the used experimental conditions.

The nickase phenotype of some of the Lid1 variants of LbCas12a described here (Fig. 3), does confirm the important role of the Lid of the RuvC domain. Together with previously described Cas12a nickases that identified involvement of the Bridge Helix, the Rec2 domain and the TNB domain, the Lid is very important in coordinating the domain rearrangements required for the sequential cleavage of the NTS and the TS. Although many new Lid variants with a nickase phenotype have been revealed in the present study (Fig. 3), the current set of mutants suggests that many residues are somehow involved in allowing the appropriate transition to the TS-cleavage state. Variation of both the size and the sequence of the Lid can be varied substantially to convert the Cas12a enzyme from a nuclease that generates DSBs (NTS and TS) in dsDNA targets into a NTS nickase. The most extreme variant of our collection is Lid2. The nickase phenotype of the Lid2 mutant is the result of a combination of two features: (i) hampered translocation of the TS toward the RuvC active site after the initial NTS cleavage and (ii) reduced overall activity. The *in vitro* analysis of target plasmid DNA reveals strong enrichment of the nicked product; however, extended reaction times also reveal appearance of linear plasmid product, indicating that the transition to the TS-cleaving state does occur, albeit at a relatively low level. The selection of Lid2 for in-depth characterization therefore reflects a balance: it combines robust nicking activity with a low, though not strictly zero, level of double-strand cleavage (Supplementary Fig. S5). Across the variants screened here, Lid2 shows one of the highest nicking scores together with one of the lowest double-strand-break scores, which is the basis for prioritizing it.

The cryo-EM structure of LbCas12a–Lid2 does reveal some of the mechanistic details that underlie its nickase phenotype, including how the engineered YCSS loop, which replaces the native α-helical Lid, forms a substrate pocket together with the RuvC catalytic residues (D832, E925, and D1172) that positions the NTS in a two-magnesium, cleavage-competent state 14 nt downstream of the PAM, with Y930 stabilizing the substrate through a hydrophobic contact analogous to F931 in the wild-type (Figs 5F and 6C–F); and how the loss of the α-helical Lid contacts with the guide–target heteroduplex (N933 with guide G9) and the Bridge Helix (E939 with R883 and R887) impairs docking of the REC2 domain, seen as an outward shift of W355 at the heteroduplex terminus (Fig. 6B and 6G–I), thereby disturbing the allosteric coupling among the Lid, REC2, and TNB domains required for target-strand loading. Complementary technical approaches, such as Molecular Dynamics analyses as well as single-molecule FRET measurements of REC2 and TNB domain dynamics, could be useful to directly probe the differential rates of TS loading between wild-type LbCas12a and the Lid2 variant. While performing this study, relevant complementary work on the Cas12a dsDNA cleavage mechanism was reported [35, 38, 61]. Building on earlier insights [60], these recent studies on LbCas12a and AsCas12a used a range of molecular analyses (biochemical, single-molecule, molecular dynamics, and cryo-EM), resulting in a complementary mechanistic model for the subsequent cleavage of the NTS and the TS by the RuvC nuclease domain of Cas12a (Fig. 6J). After PAM and seed recognition, the NTS of the dsDNA target is gradually displaced by the crRNA guide. When the R-loop extension reaches a certain level (16 bp RNA–DNA heteroduplex), the Lid of the RuvC domain adopts an α-helical conformation that forms stable interactions with nucleotides of the heteroduplex (e.g. N933 with guide-G9) as well as with amino acids of the Bridge Helix within the RuvC domain (e.g. E939 with BH-R887) (Fig. 6B). This conformational change of the Lid is a crucial event of the Cas12a catalytic mechanism in that it causes opening of the RuvC active site and it stabilizes the R-loop configuration, altogether resulting in docking of the REC2 domain with some of the terminal nucleotides of the heteroduplex. Completion of the full-length R-loop structure (20 bp heteroduplex) further stabilizes the REC2 association, allowing for appropriate positioning of the displaced NTS in the cleavage-competent RuvC catalytic site (Fig. 6A). After NTS cleavage, dissociation of the ssDNA products results in a reset of the RuvC site, and the REC2 domain is released, coinciding with partial melting of the heteroduplex (20 to 16 bp) [35, 62]; structural analyses and molecular dynamics indicate that after NTS cleavage, the Lid domain remains in the open conformation [37, 38]. Both the REC2 movement and the destabilized dsDNA configuration (with a nicked NTS) do allow for rotation and partial melting of the distal dsDNA at the TNB domain. Then REC2 docks again on the RuvC domain, while the Lid remains bound to the guide–TS heteroduplex. In addition, this domain rearrangement leads to the formation of a DNA clamp by the REC2 and TNB domains, moving the partially unwound TS to the RuvC site, eventually resulting in cleavage of the TS. The step-wise generation of a staggered dsDNA break appears to be a unique feature of DNA-targeting Cas12 nucleases (Fig. 6J) [60].

In the Lid2 variant selected in this study, the 12-residue stretch of the Lid (GFKNSRVKVEKQ) is substituted by a non-homologous 4-residue motif (YCSS). Compared to the wild-type LbCas12a, Cryo-EM analysis of the Lid2 variant shows that the disrupted Lid does not form an α-helical conformation and, consequently, that it does not interact with the guide–target heteroduplex (Fig. 6D). Whereas F931 of the wild-type α-helical Lid stabilized the NTS in the RuvC active site, the Y930 residue in this YCSS motif of the Lid2 variant allows for an analogous interaction that appropriately positions the NTS for cleavage by the RuvC catalytic site (Fig. 6B and D). Moreover, the loss of interactions between the α-helical Lid and the guide–target heteroduplex results in some conformational changes in the docking of the REC2 domain (W355 and 20th base pair of heteroduplex) (Fig. 6HI). Apparently, the slightly disturbed REC2 docking also affects subsequent mechanistic events after NTS cleavage, including REC2 release and subsequent TNB domain rearrangement. This would explain why LbCas12a–Lid2 is hampered in adopting a TS-clamping state, significantly impairing TS cleavage while retaining robust NTS cleavage.

All Cas12 and related TnpB-like enzymes possess a single RuvC-like nuclease domain [60]. Using a similar catalytic mechanism, cleavage of dsDNA targets generates sticky ends with 5′ overhangs [9]. The first characterized enzyme FnCas12a was demonstrated to cleave its target dsDNA after the 18th PAM-distal nucleotide on the NTS, and after the 23rd nucleotide on the TS. When testing different targets, minor variations in the location of the cleavage sites were found [9]. Likewise, similar cleavage patterns were revealed in case of other Cas12 enzymes, however often with variable cleavage sites, resulting in sticky ends with overhangs varying from 2 to 10 nt. The cleavage pattern can also be slightly influenced by the length of the spacer [63]. Our data on LbCas12a reflect the Cas12a cleavage pattern, including the site and overhang heterogeneity. Sanger sequencing of the nicked product resolves the Lid2 NTS cleavage reproducibly at position 14, that is, 14 nt downstream of the PAM (Fig. 4B). The wild-type LbCas12a NTS cleavage position was not resolved in the present study; for the prototypical enzyme, the Cas12a NTS cut site was resolved by Sanger sequencing of the cleavage products in earlier work [9]. This position is consistent with the presented structure of Lid2, in which the scissile phosphate between nucleotides 14 and 15 is positioned in a cleavage-compatible state in the RuvC active site (Fig. 5F). Additional mapping of cleavage and nicking events also spans positions 14–18, indicating that NTS scission is variable and yields heterogeneous 5′ overhangs [37]. Mechanistic studies further show that NTS cleavage is required for TS loading: a 14-bp RNA–DNA hybrid activates Cas12a but cannot expose the native NTS site; TS cleavage is restored only when the NTS is pre-nicked at its physiological position, and pre-nicking the TS does not trigger NTS cutting [56]. This implies that, in case of wild-type Cas12a enzymes, NTS cleavage within a permissive window of 14–18 nt downstream of the PAM is required for positioning the TS in the active site. Hence, the Lid2 cut at position 14 falls within the 14–18 window of natural variability of Cas12a effectors.

The successful engineering of an RNA-guided Cas12a variant with a robust nickase phenotype is an addition to the genome editing toolbox, potentially allowing for different editing approaches. First, the use of a Cas12a nickase pair with two guides is a way to increase the specificity of genome editing, as has been reported for Cas9 nickases [10]. In case of nCas12a, a short synthetic CRISPR array may be used, relying on the intrinsic guide processing capacity of Cas12a [64]. Second, using Cas9 variants to introduce site-specific nicks in genomic DNA of eukaryotic cells generally results in generation of random mutations (indels) or in homology-directed recombination (HDR) [20, 23]. Again, the advantage of using nickase variants instead of their wild-type counterparts may be the fact that DSBs are avoided, lowering the risk of unwanted chromosome rearrangements. Third, nicking of the NTS may allow for prime editing. A first generation of prime editors has been based on NTS-nicking variants of Cas9, either with a fused reverse transcriptase (RT) domain [31] or with a split RT domain [65]. Upon target recognition and nicking of the NTS, the 3′ end of the nicked strand can base pair with a complementary 3′ extension of the pegRNA. Next, the RT uses the central part of the pegRNA as template to extend the nicked NTS to introduce the desired edits as well as part of the original NTS. The later stretch allows for base pairing with the TS, and eventually in recombination to introduce the desired edit [31].

A first example of a Cas12a-based prime editor is the Redraw design in which a wild-type Cas12a is fused to an RT domain. After generating a double strand DNA break, an extension of the pegRNA can, downstream the generated break in the TS, base pair with the 3′ end of the TS. Next, the RT uses the editing design template in the central part of the pegRNA to regenerate the TS including the desired edits that will also be introduced in the target DNA after recombination [66]. Based on the nickase activity of LbCas12a (R1138A) [15], also different prime editors has been developed [67]. The Cas12 nickase is fused to the RT as well as to the MS2 coat protein (MCP). Targeting occurs with a small guide, whereas a second, circular MS2–pegRNA is responsible for the prime editing of the nicked NTS. The MCP domain is responsible for recruiting the MS2 part of the pegRNA. Efficient prime editing in human cells has been reported. In the present study, the nickase activity of LbCas12a R1138A is compared to that of the LbCas12a Lid2 variant (Fig. 1D). The fact that, at least under the tested *in vivo* conditions, the Lid2 acts as a relatively robust nickase, makes this variant a promising candidate for the future development of novel Cas12a prime editors. Indeed, the Lid2 variant has recently served as the basis for an efficient Cas12a-based prime editor in both plant and human cells, with very low levels of off-targeting. This work provides an experimental demonstration of utility and confirms that the uncoupling of NTS and TS cleavage achieved by the Lid2 remodeling supports prime editing in eukaryotic cells (De Vleesschauwer *et al*., under revision).

We originally designed the screening strategy to interrogate an NNK-based saturation mutagenesis library. However, the isolation of the Lid2 deletion variant offers mechanistic insight into the plasticity of the Lid domain. The screen identified a functional solution where a flexible loop replaces the ordered α-helical Lid. This alteration effectively uncouples the mechanism required for TS cleavage while maintaining NTS activity. These findings complement recent studies and suggest that future engineering efforts for Cas12a nickases should explore insertions and deletions that reshape domain interfaces rather than relying solely on single-residue substitutions, as gross structural remodeling of the Lid appears necessary to fully uncouple the two cleavage steps. Apart from the Lid2 variant, numerous other LbCas12a nickase variants with a range of deletions and/or substitutions in the Lid domain have been generated in the present study, including stepwise C-terminal and N-terminal Lid truncations and saturation-mutagenesis libraries at defined Lid positions. Beyond the variants described here, it is anticipated that many more Cas12a–Lid variants with nickase activity either exist in nature or can be generated in the lab. More generally, a robust nicking activity in bacteria or *in vitro* may not be a prerequisite for efficient nicking in a eukaryotic gene-editing context. A slower second-strand cleavage that accumulates a nicked intermediate can already provide a sufficient phenotype, which is why this study sought a nickase phenotype, defined by the accumulation of nicked DNA, both to probe the cleavage mechanism and to identify variants of potential utility as editing tools.

## Supplementary Material

gkag934_Supplemental_Files

## Data Availability

The cryo-EM density map and atomic coordinates for the LbCas12a–Lid2–crRNA–target DNA complex have been deposited in the Electron Microscopy Data Bank (EMDB) and Protein Data Bank (PDB) under accession codes EMD-66115 and 9WNE, respectively (https://doi.org/10.2210/pdb9WNE/pdb). Selected plasmids will be deposited at Addgene. All other data supporting the findings of this study are available from the corresponding author at Wageningen University & Research upon reasonable request. During the preparation of this manuscript, the authors used OpenAI’s ChatGPT to assist with stylistic editing and language refinement. After using this tool, the authors reviewed and edited the content when needed. The authors take full responsibility for the content of the final manuscript.

## Notes

Present address: GeneTwister, Nieuwe Kanaal 7b, 6709 PA Wageningen, Netherlands

Present address: Chemical and Pharmaceutical Biology, Groningen Research Institute Pharmacy, Groningen University, 9713 AV Groningen, Netherlands
