# Supplementary material for: Size and sequence variation of the Lid of the Cas12a nuclease domain results in a nickase phenotype
Source: Nucleic Acids Res. 2026 Oct 5;54(18):gkag934. doi: 10.1093/nar/gkag934 (PMC13635745; doi:10.1093/nar/gkag934)
Supplement: Supplementary file 1 [file gkag934_supplemental_files.zip › 04_Supplementary_Information_NAR-00343_SO.pdf]

Supplemental Information

Size and sequence variation of the Lid of the Cas12a nuclease domain results in a nickase phenotype

Ricardo Villegas Warren, Satoshi N. Omura, Diana Leon Castro, Christian Südfeld, Maartje Luteijn, Hayato Morinaga, Wakana J. Inoue, Yutaro Shuto, Anne-Marie Kuijpers, Frank Meulewaeter, David De Vleesschauwer, Raymond H.J. Staals, Osamu Nureki, Wen Y. Wu, John van der Oost*

- **Supplementary Figures**
    - Figure S1. Cloning artefact resulting in the Lid2 variant
    - Figure S2. Reporter-screen kinetics
    - Figure S3. N-terminal Lid trimming series
    - Figure S4. GFP/RFP phenotype map of the LbCas12a Lid variants
    - Figure S5. In-vitro cleavage of Lid2: time course, MgCl2 titration gel, and quantification
    - Figure S6. Cryo-EM analysis of the LbCas12a-Lid2 crRNA-target DNA ternary complex
    - Figure S7. Representative cryo-EM densities
    - Figure S8. Alignment of 16 Cas12a orthologs
    - Figure S9. Fluorescent reporter plasmids
- **Supplementary Tables**
    - Table S1. Cloning primers
    - Table S2. Plasmid sequences used in this study
    - Table S3. Relevant sequences used in this study
    - Table S4. Data collection, processing, model refinement, and validation
    - Table S5. Engineered Lid variants: Lid sequence and nicking phenotype
    - Table S6. Oligonucleotides used in the lid trimming assay
    - Table S7. Per-position Sanger base calls and base-call quality across the LbCas12a-Lid2 non-target-strand cleavage site
- **References**

Supplementary Figure 1. Cloning artefact resulting in the Lid2 variant.

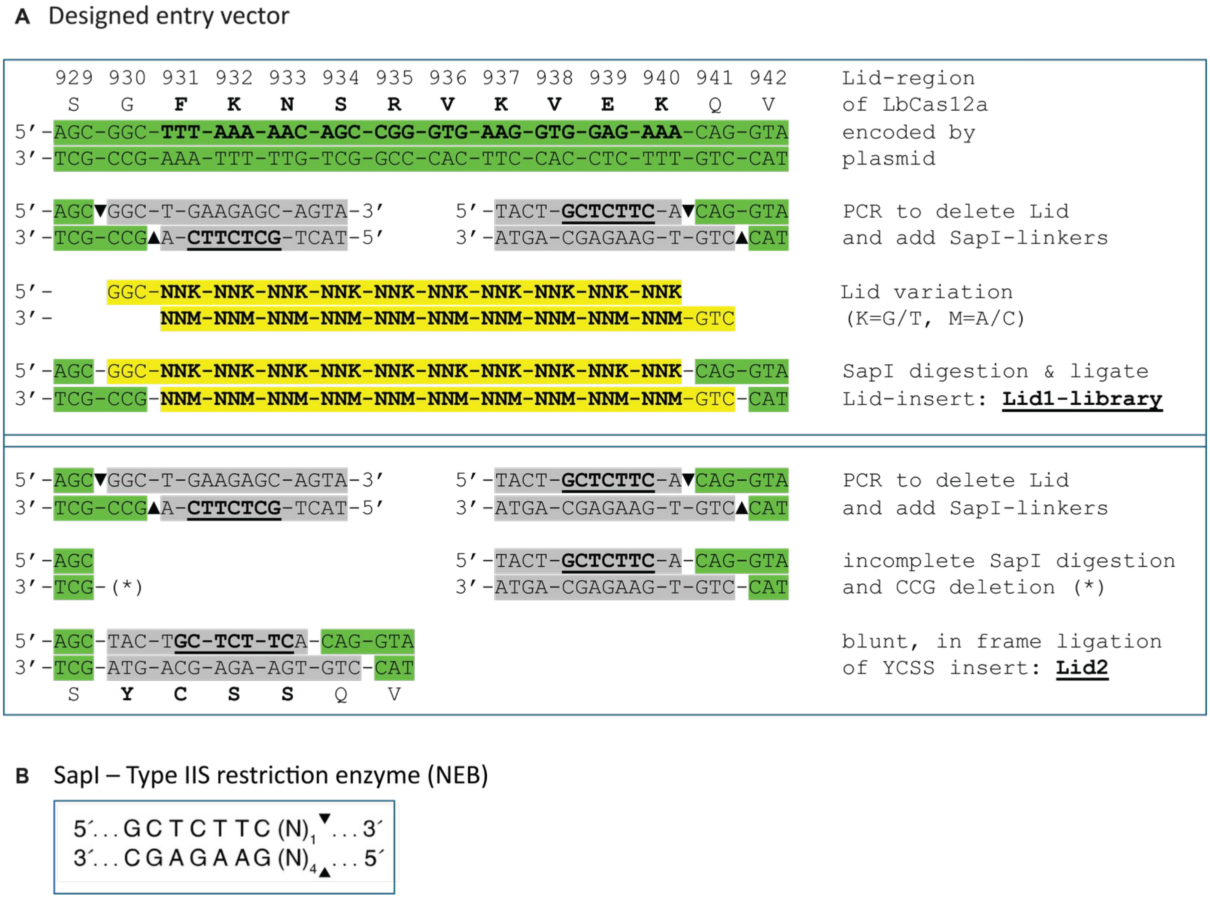

Supplementary Figure 1. Cloning artefact resulting in the Lid2 variant. (A) Designed SapI-based entry vector for saturation mutagenesis of the LbCas12a Lid. The native Lid-region sequence (residues 929 to 942) is excised by PCR and flanked with SapI recognition sites, so that SapI digestion and ligation directionally insert a degenerate NNK-10 cassette (K = G/T, M = A/C) to build the Lid1 substitution library (top). The Lid2 variant arose as a cloning artefact of the same reaction (bottom): incomplete SapI digestion (only one linker site cleaved), together with a CCG deletion (asterisk), left blunt-compatible ends that re-ligated in frame to encode the short YCSS loop of Lid2. **(B)** SapI is a Type IIS restriction enzyme (NEB) that recognizes GCTCTTC and cleaves one and four nucleotides downstream of its recognition site, leaving a three-nucleotide 5' overhang.

Supplementary Figure 2. Reporter-screen kinetics.

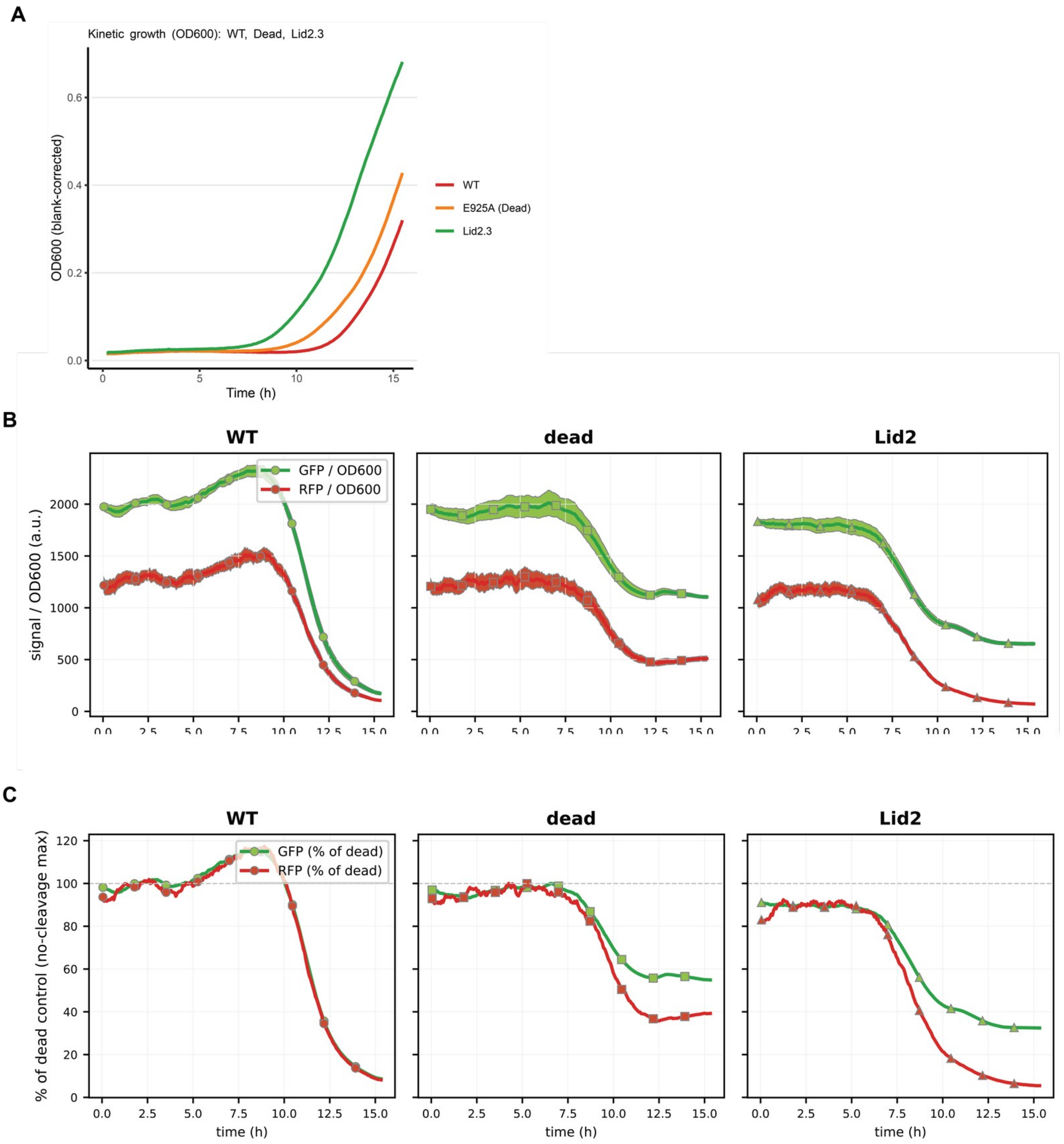

Supplementary Figure 2. Reporter-screen kinetics. Continuous plate-reader time course (about 15 h) of the dual-fluorescent reporter strain expressing wild-type LbCas12a (WT), the catalytic-dead E925A control (Dead), or Lid2. (A) Blank-corrected OD600 growth curves. **(B)** GFP and RFP fluorescence per OD600 over time for each strain (mean with shaded error band across biological replicates). (C) The same signals expressed as a percentage of the catalytic-dead (no-cleavage) control over time. Wild-type LbCas12a depletes both reporters, the dead control retains both, and Lid2 shows the GFP+/RFP- nickase signature, with RFP falling below GFP.

Supplementary Figure 3. N-terminal Lid trimming series.

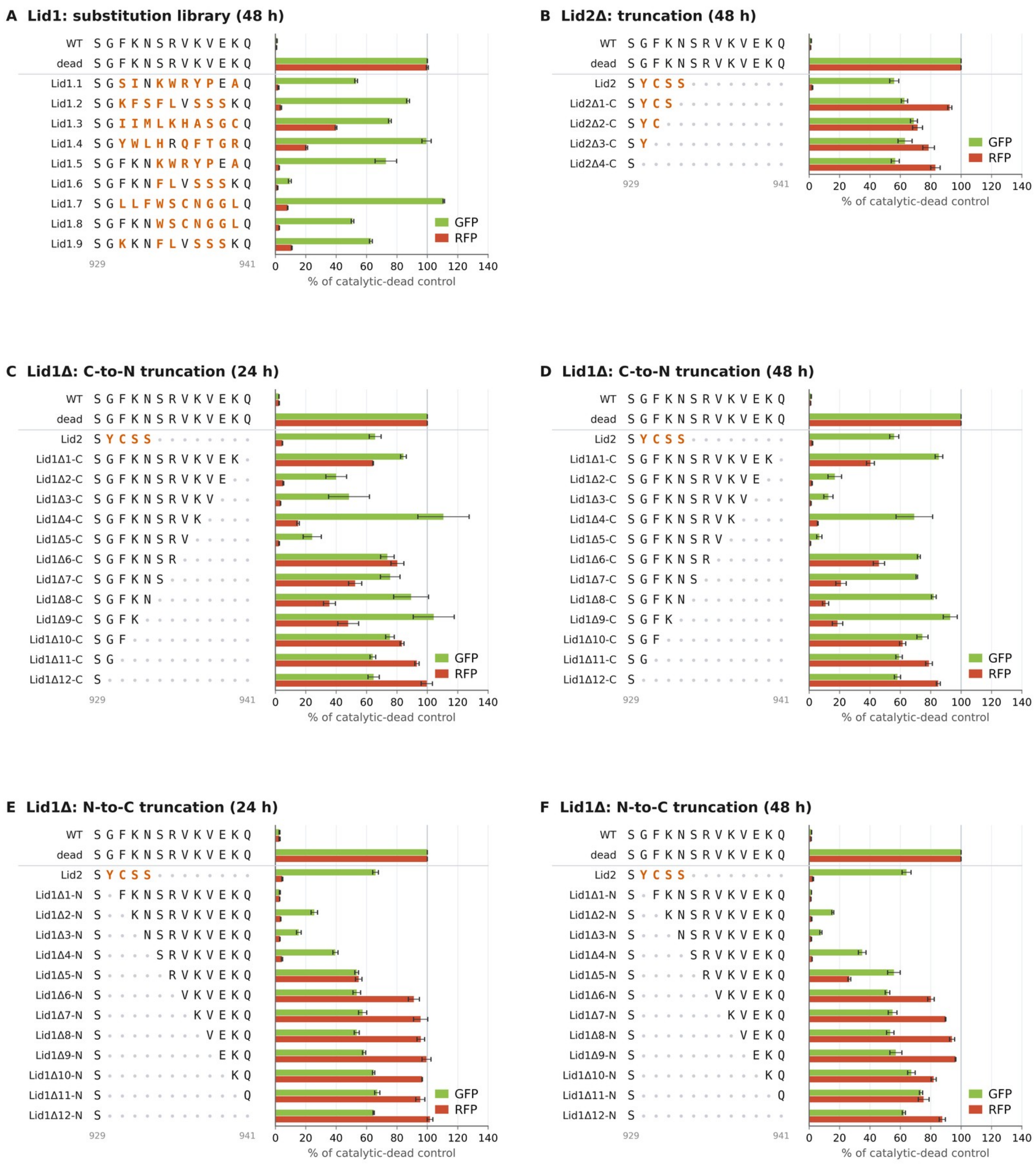

Supplementary Figure 3. Dual-fluorescent reporter activity of the Lid variant panel at two culture time points and in both Lid-truncation directions. Engineered LbCas12a Lid variants were screened in the dual-fluorescent reporter assay against wild-type LbCas12a and the catalytic-dead D832A/E925A control, as in Figure 3. Each panel shows GFP and RFP reporter signal as a percentage of the catalytic-dead control (mean and SEM of three biological replicates) above the matched 929 to 941 Lid amino-acid alignment. Two time points are compared throughout: 24 h refers to the first overnight culture (the reading shown in Figure 3) and 48 h to a second reading taken after re-inoculation and overnight incubation. **(A)** Lid1: the NNK-10 substitution library (Lid1.1 to Lid1.9), corresponding to the variants in Figure 3A

at the 48 h time point. **(B)** Lid2Δ: the Lid2 (YCSS) motif progressively truncated (Lid2 through Lid2Δ4-C), corresponding to the variants in Figure 3D at the 48 h time point. **(C, D)** Lid1Δ: C-to-N truncation of the native Lid (Lid1Δ1-C to Lid1Δ12-C) at 24 h (C, top; the same data as Figure 3B) and 48 h (D, bottom). **(E, F)** Lid1Δ: N-to-C truncation of the native Lid (Lid1Δ1-N to Lid1Δ12-N) at 24 h (E, top) and 48 h (F, bottom). The number in each truncation name counts residues deleted: for the Lid1Δ variants from the native Lid, and for the Lid2Δ variants from the four-residue YCSS motif, so that Lid2Δ4-C corresponds to the complete YCSS deletion.

Supplementary Figure 4. GFP/RFP phenotype map of the LbCas12a Lid variants.

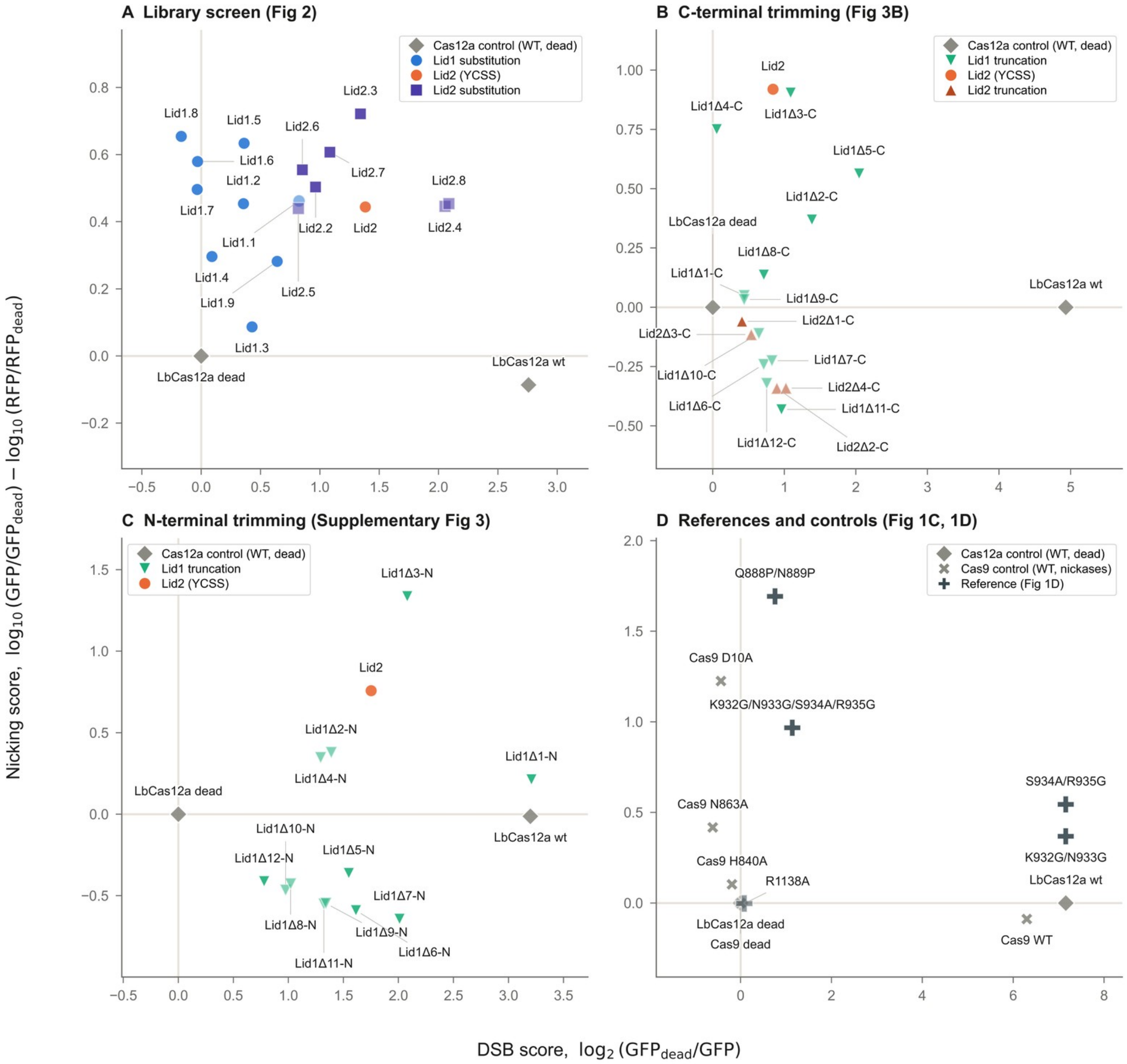

Supplementary Figure 4. GFP/RFP phenotype map of the LbCas12a Lid variants. Each variant is positioned by its dual-fluorescent reporter signal relative to the catalytic-dead control: the DSB score is log2(GFPdead/GFP), so higher values indicate more double-strand cleavage, and the nicking score is log10(GFP/GFPdead) minus log10(RFP/RFPdead), so higher values indicate more nicking (GFP retained relative to RFP). A control whose reporter is fully cleaved (below detection) is placed at a nicking score of zero, as a balanced double-strand cutter has no strand preference. Each panel corresponds to one independent plate read and is normalized to that plate's own catalytic-dead control, which sits at the origin, so scores are compared within a panel rather than across panels. (A) The Lid saturation-mutagenesis screen (Figure 2): the Lid1 and Lid2 substitution series against wild-type LbCas12a and the dead control. (B) The C-terminal Lid truncation series (Figure 3B). (C) The N-terminal Lid truncation series (Supplementary Figure 3). (D) Reference points from separate experiments: the SpCas9 controls (Figure 1C) and the previously described LbCas12a variants (Figure 1D), comprising Q888P/N889P, R1138A, the K932G/N933G/S934A/R935G quadruple, and the two double mutants. Colour and marker shape both denote the variant group. Wild-type LbCas12a and SpCas9 sit at high DSB and low nicking, and the SpCas9 D10A nickase at negative DSB and high nicking. The Lid2 (YCSS) variant appears in each panel in which it was measured (A, B and C),

illustrating that a variant reads differently across plates; its main-text characterization uses the Figure 3B read, where it combines a robust nicking score with a low but non-zero DSB score. Values are per-plate ratios to the dead control.

Supplementary Figure 5. In-vitro cleavage of Lid2: time course, MgCl2 titration gel, and quantification.

A

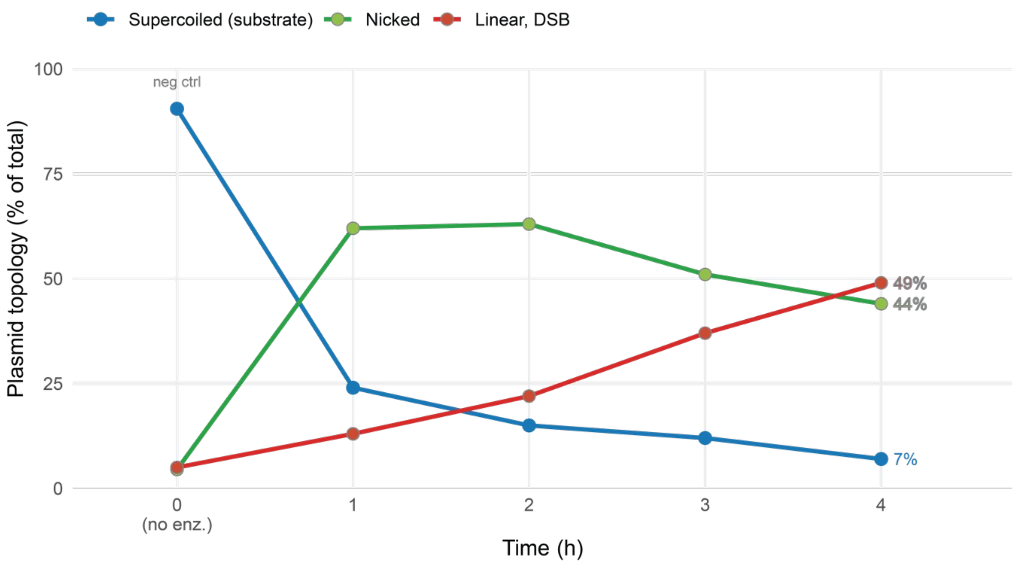

B

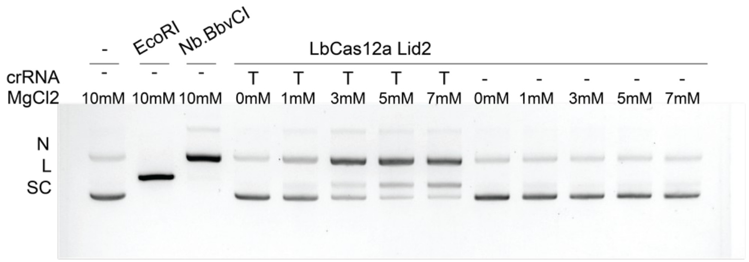

C

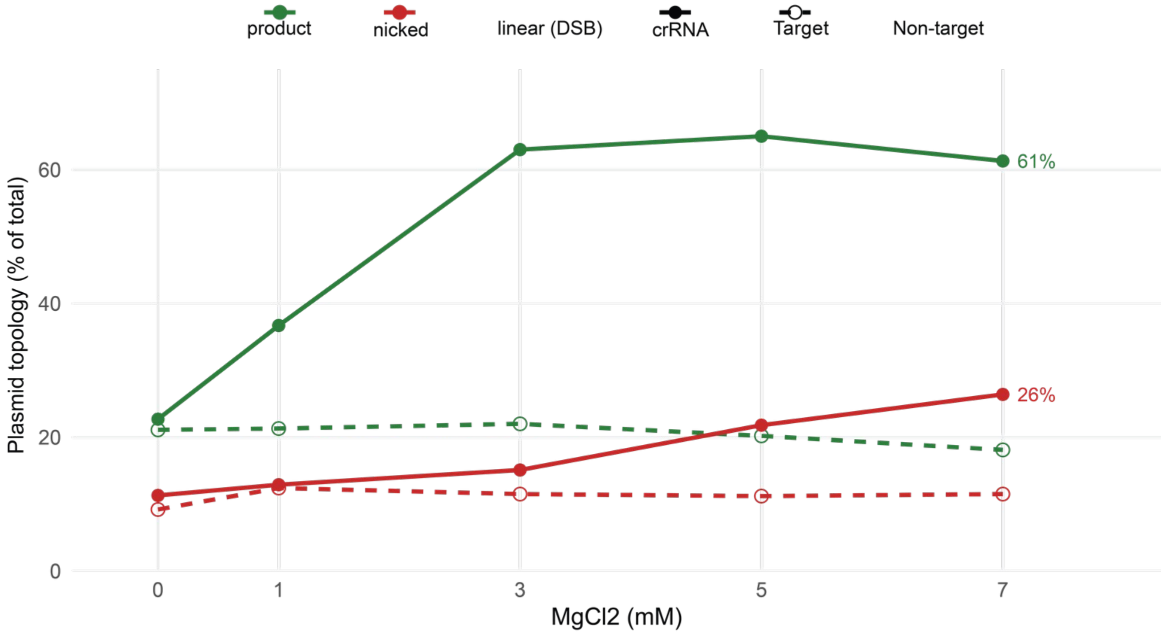

Supplementary Figure 5. In-vitro cleavage of Lid2: time course, $MgCl_2$ titration gel, and quantification. **(A)** Time-course quantification of the LbCas12a-Lid2 cleavage assay: supercoiled, nicked and linear (double-strand-break) fractions of pRV013 over time (t=0 is the no-enzyme control); this quantifies the gel shown in main Figure 4C. **(B)** Agarose gel of the MgCl2 titration: supercoiled pRV013 cleaved by LbCas12a-Lid2 with the targeting (T) or non-targeting (-) crRNA across 0 to 7 mM MgCl2, alongside no-enzyme, EcoRI and Nb.BbvCI controls (N, nicked; L, linear; SC, supercoiled). The restriction-enzyme controls were run in CutSmart buffer; the LbCas12a-Lid2 reactions used the cleavage buffer at the indicated MgCl2 concentrations. **(C)** Per-lane densitometry of the gel in B; nicking rises with MgCl2 for the targeting crRNA while the non-targeting control stays flat and double-strand cleavage stays minor.

Supplementary Figure 6. Cryo-EM analysis of the LbCas12a-Lid2 crRNA-target DNA ternary complex.

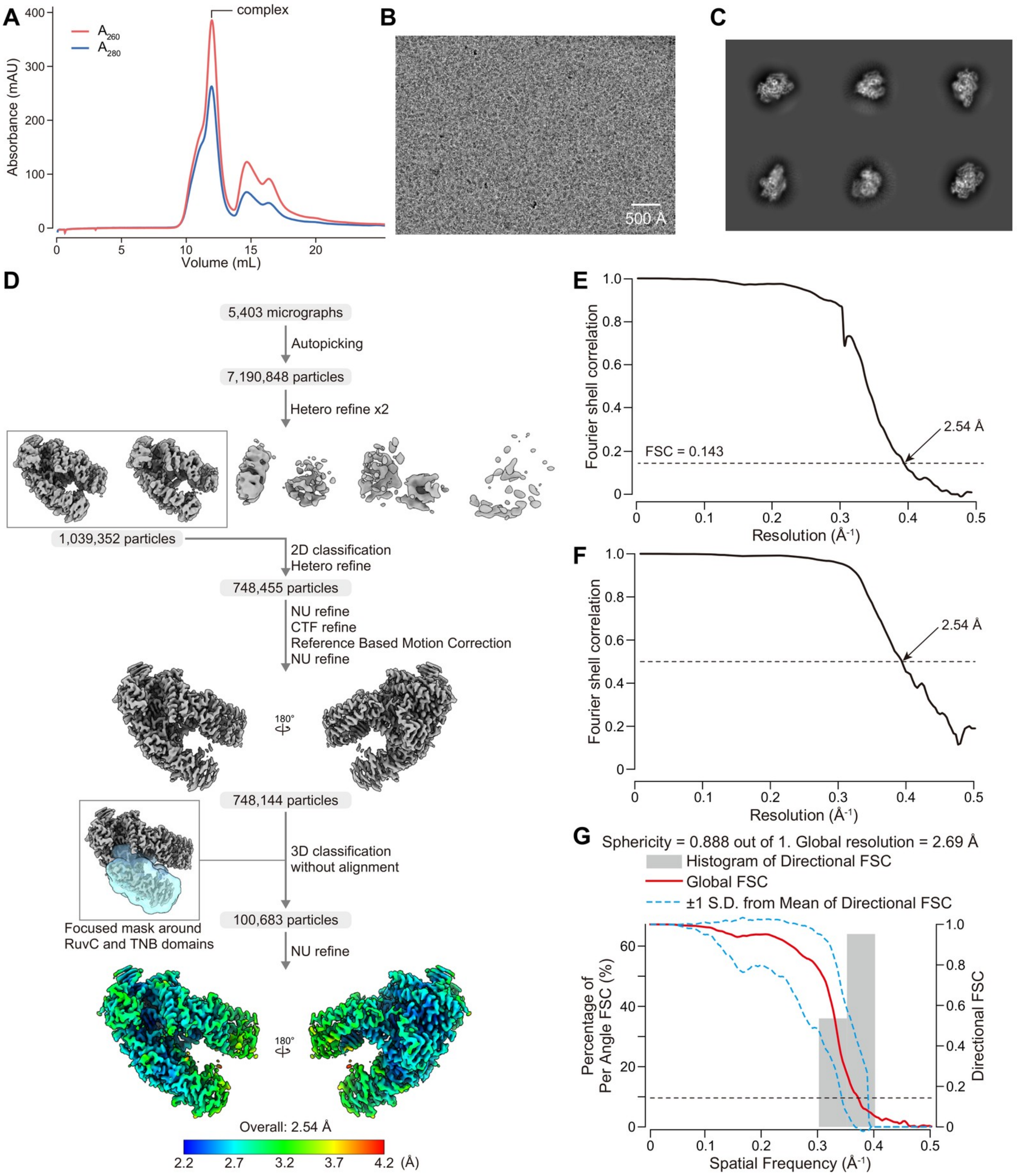

Supplementary Figure 6. Cryo-EM analysis of the LbCas12a-Lid2 crRNA-target DNA ternary complex. (A) Size-exclusion chromatography profile of the LbCas12a-Lid2 crRNA-target DNA complex; the peak fraction indicated as complex was used for cryo-EM. **(B)** Representative cryo-EM micrograph, recorded on a 300 kV Titan Krios with a K3 camera. **(C)** Representative 2D class averages. **(D)** Single-particle image-processing workflow (cryoSPARC v4.4.0), with density maps colored by local resolution shown at the bottom. (E) Fourier shell correlation (FSC) curve of the final reconstruction; the FSC = 0.143 criterion indicates an overall resolution of 2.54 Å. (F) Model-to-map FSC curve; the FSC = 0.5 criterion indicates a resolution of 2.54 Å. (G) Directional FSC analysis of the final map, with a sphericity of 0.888 and a global resolution of 2.69 Å.

Supplementary Figure 7. Representative cryo-EM densities.

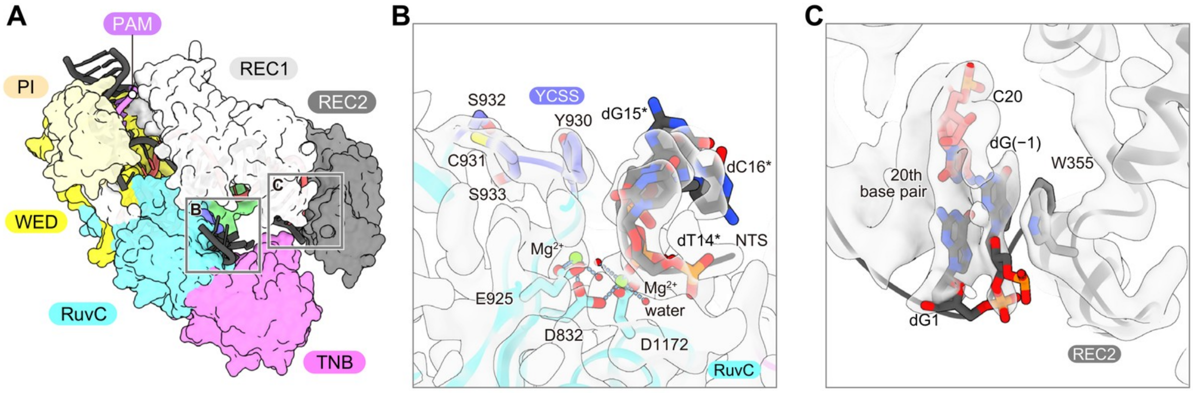

Supplementary Figure 7. Representative cryo-EM densities. (A) Surface representation of the LbCas12a-Lid2 crRNA-target DNA complex. **(B, C)** Close-up views of the RuvC active site **(B)** and the terminus of the guide-target heteroduplex **(C)**; cryo-EM density maps are shown as grey semi-transparent surfaces, with the bound Mg2+ ions and water molecules as light green and red spheres, respectively.

Supplementary Figure 8. Alignment of 16 Cas12a orthologs.

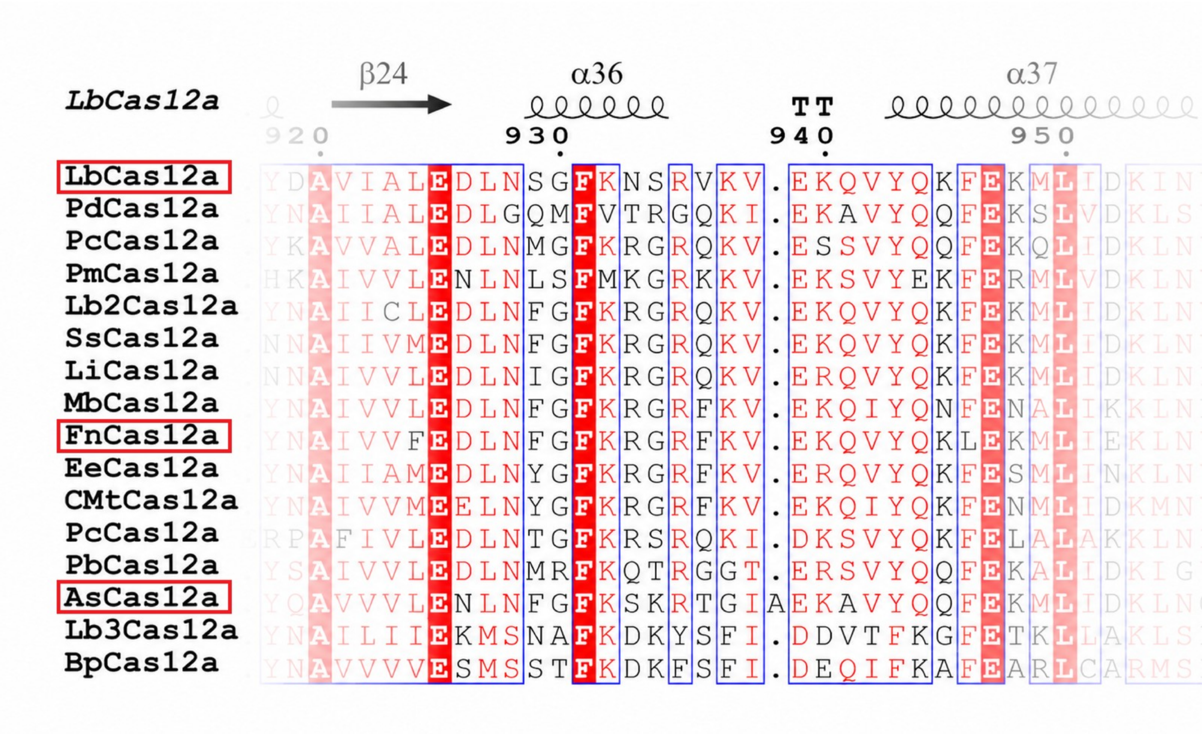

Supplementary Figure 8. Amino acid Lid-region alignment of 16 Cas12a orthologs. LbCas12a structure and numbering; LbCas12a, FnCas12a and AsCas12a representatives are boxed in red; conserved positions are boxed in blue. Detail of the Lid region (residues 920 to 950): K932 and the basic residue R935 are conserved while N933 and S934 are divergent, the positions that define the Lid2 nickase motif.

Supplementary Figure 9. Fluorescent reporter plasmids.

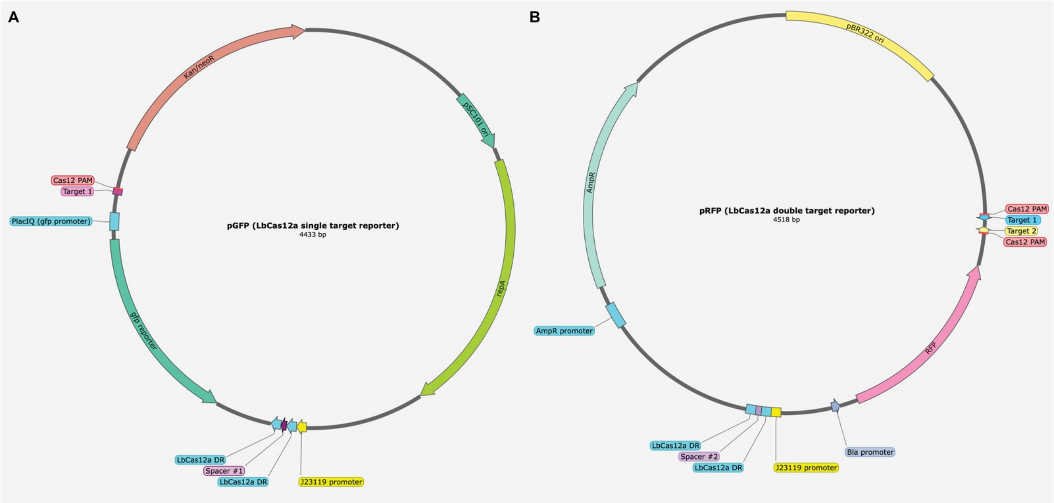

Supplementary Figure 9. Fluorescent reporter plasmids. (A) pGFP, the single-target reporter, combines a pSC101 origin of replication with its repA gene and a kanamycin resistance marker (Kan/neoR) with the gfp reporter gene expressed from the PlacIQ promoter. A single LbCas12a target site (Target 1, protospacer PS1 preceded by a TTTA PAM) is located in the non-coding region immediately upstream of the gfp promoter. The crRNA cassette is encoded on the reporter plasmid itself: Spacer #1, which matches Target 1, is flanked by two LbCas12a direct repeats (DR) and expressed from the J23119 promoter. **(B)** pRFP, the double-target reporter, combines a pBR322 origin of replication and an ampicillin resistance marker (AmpR) with the rfp reporter gene expressed from the bla promoter. Two adjacent target sites in opposite orientation (Target 1 and Target 2, protospacers PS1 and PS2, each preceded by a TTTA PAM and separated by 24 bp) are located in the non-coding region downstream of the rfp gene, and the crRNA cassette encoded on this plasmid carries Spacer #2, which matches Target 2. Target 1 is identical in both reporters whereas Target 2 is unique to pRFP, so that in the dual-reporter strain a nickase variant introduces a single nick in pGFP but paired nicks on opposite strands in pRFP; paired nicking is expected to generate a double-strand break and loss of the RFP reporter, which is the basis of the green (GFP-positive, RFP-negative) nickase phenotype (Figure 1A). Because the target sites are located in non-coding sequences, cleavage leads to loss of the reporter plasmid rather than to an indel in gfp or rfp. The annotated maps of both plasmids are listed in Supplementary Table S2, and the primer, spacer and target sequences are listed in Supplementary Tables S1 and S3.

Supplementary Table S1: Cloning primers

Name	Seq	BG code
**Cloning guide gfp Esp3I rv**	ATACTCGTCTCTTCCGCTGAGCAATAACTAGCATAACC	BG25640
**Cloning guide gfp Esp3I fw**	ATACTCGTCTCTATCTACACTTAGTAGAAATTATTTAATCTTTGAAACGCTAGCATTATACCTAGGACTGAGC	BG25639
**pGFP insert SP1 DR1 fw**	CGGAATCTACACTTAGTAGAAATTATTTAATCTTTGAAACAGGGTAAGGTTTCCCATCGT	BG25641
**pGFP insert SP1 DR1 rev**	AGATACGATGGGAAACCTTACCCTGTTTCAAAGATTAAATAATTTCTACTAAGTGTAGAT	BG25642
**Cloning guide rfp BbsI Fw**	TACTGAAGACTGCCGGCTTATCGGTCAG	BG25643
**Cloning guide rfp BbsI rv**	TACTGAAGACTAATCTACACTTAGTAGAAATTATTTAATCTTTGAAACGCTAGCATTATACCTAGGACTGAGC	BG25644
**Insert guide2 in rfp fw**	AGATCTCCACTGAACCTGGGGAATGTTTCAAAGATTAAATAATTTCTACTAAGTGTAGATTTTTTTTG	BG25645
**Insert guide2 in rfp rv**	CCGGCAAAAAAAATCTACACTTAGTAGAAATTATTTAATCTTTGAAACATTCCCCAGGTTCAGTGGAG	BG25646
**NNK library insert Fw**	AGCGGCNNKNNKNNKNNKNNKNNKNNKNNKNNKNNKCAG	BG25836
**NNK libraries insert Rv**	TACCTGMNNMNNMNNMNNMNNMNNMNNMNNMNNMNNGCC	BG25837
**pCas LbCas12a SapI LidFw**	TACTGCTCTTCCGTATACCAAAAGTTTGAAAAGATGCTCATTG	BG25788
**pCas LbCas12a SapI LidRV**	TACTGCTCTTCCGCTATTGAGGTCTTCCAGG	BG25789
**Protospacer_Cas12a_puc19 oligo FW (pRV013)**	GATCCTCAGCTTTAGTCGAGTGCAAAACCTTTCGCGGTATGGCAACATCTGTACGTAGCATTCG	BG25126
**Protospacer_Cas12a_puc19 oligo REV**	AATTCGAATGCTACGTACAGATGTTGCCATACCGCGAAAGGTTTTGCACTCGACTAAAGCTGAG	BG25127
**LbCas12a Cy3 target Fw**	GGTAGAGGATCCTCAGCTTTAGTCGAGTGCAAAACCTTTCGCGTAGCATTCGAATTCACC	BG27844
**LbCas12a Cy5 target Fw**	GGTGAATTCGAATGCTACGCGAAAGGTTTTGCACTCGACTAAAGCTGAGGATCCTCTACC	BG27845
**pUC19 Target seq Rv**	ATATGCGGTGTGAAATACC	BG33284
**pUC19 Target seq Fw**	CTGGCACGACAGGTTTCC	BG33285

Supplementary Table S2. Plasmid sequences used in this study

Plasmids name	Plasmid Map	Source
**pCas entry vector**	https://benchling.com/s/seq-KErLPU7dsmho9kbmdDoJ?m=slm-QASylUHnsu4ySfynyeQn	Wu et al[2]
**pGFP entry vector**	https://benchling.com/s/seq-U4QqemR87fr4fPIlhBoB?m=slm-6pcmwT5zEM4KaamZP2mw	Wu et al[2]
**pRFP entry vector**	https://benchling.com/s/seq-cTRBUDaScAbZtsS3mMlT?m=slm-UqMTz54hJC5NcTWkMci7	Wu et al[2]
**pCas LbCas12a wt**	https://benchling.com/s/seq-vzQDRBqUXTQbT7WfK8aP?m=slm-0RSsRlxYxeXfNsbry8e5	This study
**pCas LbCas12a dead**	https://benchling.com/s/seq-B1b8IrawZoM9XDa9cYyJ?m=slm-Ogg5oljdbR500Z36ewZd	This study
**pRV067_pCas_-_LbCas12a_K932GN933G_PJ23108_-_pCB482_NA**	https://benchling.com/s/seq-6pkwibg6oAyiK8wV5O7A?m=slm-ysnfvAQ0oZq6EzAxaCaO	This study
**pRV068_pCas_-_LbCas12a_K932GN933GS934AR935G_PJ23108_-_pCB482_NA**	https://benchling.com/s/seq-blhEGc6cGWr3h8fHMvas?m=slm-vFpX6lSu3WzD1wQWKjkG	This study
**pRV069_pCas_-_LbCas12a_S934AR935G__PJ23108_-_pCB482_NA**	https://benchling.com/s/seq-MR0SvyddcZ0q7tRwGYGp?m=slm-JVNyo7GLZlBLmEDR9JhY	This study
**pCas LbCas12a Lid2**	https://benchling.com/s/seq-ZioFLDk9VIeUcdQtMvF3?m=slm-m852ffmExfaTGovpersf	This study
**pCas LbCas12a Lid NNK saturation library 25836**	https://benchling.com/s/seq-kZ4hFK1VlLgyUJmGFG35?m=slm-ExbKVbkUS1AeLtNKz7RR	This study
**pCas SpCas9 wt**	https://benchling.com/s/seq-M2IUJwHlISLWISewrpaY?m=slm-PFV04Q0DJbWIfX7PcKgn	This study
**pCas SpCas9 D10A**	https://benchling.com/s/seq-lThgfvRUPLxDiZQ6ZRiI?m=slm-n1zv9cj2s7aSRSUeeDGD	This study
**pCas SpCas9 H840A**	https://benchling.com/s/seq-dARxrtKQumQM7MgfNxgt?m=slm-	This study

	T7vhiC6xV4zBxAkWWRSU	
**pRV045_pCas_N863A_-_eCas9_PJ23108_-_pCB482_NA**	https://benchling.com/s/seq-ddKprc4Pu1QLJ1vsb9xM?m=slm-MtXmvKMvSUT9frH4FP7d	This study
**pCas SpCas9 D10A/H840A**	https://benchling.com/s/seq-YlfqsbXvFfdX2uzPXnV7?m=slm-3nDlwQKO0cbSvSOeMXVj	This study
**pGFP SpCas9 (PS1, Guide1)**	https://benchling.com/s/seq-xiBR7lwY4J84MhXxUSda?m=slm-rSHTTCUWzlDzSwsmQJ4K	This study
**pRFP SpCas9 (PS1, PS2, Guide2)**	https://benchling.com/s/seq-si6nCTn5xYwpRTM3ZQeq?m=slm-8XDASGSJ9CrX634C596a	This study
**pGFP LbCas12a (PS1, Guide1)**	https://benchling.com/s/seq-XOtnTdAktboMjaktsfy0?m=slm-yRqOmqwJVBKkSUrsZpZn	This study
**pRFP LbCas12a (PS1, PS2, Guide2)**	https://benchling.com/s/seq-QYasYcv9B2z6nebxfjzW?m=slm-JVUjMnox4C1ovzvmzQpb	This study
**pET-His6-MBP-TEV-LIC (1M) cloning vector**	https://benchling.com/s/seq-T9yPWCxk6g3OVs1nrT2v?m=slm-g46Iqj79H8hoVEByqeWx	Swarts et al, 2017[3]
**pET His(6)-LbCas12a wt**	https://benchling.com/s/seq-jc87rilovrgyV0W3qf7y?m=slm-CxshxuUF4JquLAd1Pl1f	This study
**pET His(6)-LbCas12a D1180A**	https://benchling.com/s/seq-q6THUhPqYK8Cghgd5gLB?m=slm-p27UyX5CV0b9gJb2CEIw	This study
**pET His(6)-LbCas12a Lid2 (table S1)**	https://benchling.com/s/seq-QTc28QxLgeNLZ433xMX6?m=slm-Pk5dfJlGkpgQCrz4SDLF	This study
**pET His(6)-LbCas12a R1138A**	https://benchling.com/s/seq-lFLeM3ToW1JYWLNypgyr?m=slm-SMaPC6XRMA0PyH6eSBNs	This study
**pRV024_LbCas12a_Q888PN889P__BH_pET**	https://benchling.com/s/seq-eePLC20WwnchbmdrVLOQ?m=slm-PUv5SAbM4JHi4lMJK9iy	This study
**pUC19 entry vector**	https://benchling.com/s/seq-l2KavNFtM2DFHzxFvvnX?m=slm-uUnTcpTDwpAvtOyOiuVk	
**pRV013 Target**	https://benchling.com/s/seq-	This

	At1wPUi7EKHkn9L4P6NP?m=slm-g74AjTeXEpCA2L50L9RV	study
**pRV350 LidΔ11**	https://benchling.com/s/seq-EKB1beRbwMbslCvchSyy?m=slm-Tput9C6fByT5lieE6yum	This study
**pRV351 LidΔ10**	https://benchling.com/s/seq-dhm1spwxWm8cAtW3ZPxt?m=slm-M74C65FU3mnUG0MoAvCh	This study
**pRV352 LidΔ9**	https://benchling.com/s/seq-q8vJYcwV1OZEqRNZdGzy?m=slm-IlNBN89vvhUfJpK0oifT	This study
**pRV353 LidΔ8**	https://benchling.com/s/seq-7DbPrnxjBRTUQIA02stk?m=slm-T5vKnNkto0VhJMVXYb91	This study
**pRV354 LidΔ7**	https://benchling.com/s/seq-gTdW0OQzN7Xgs4mrVLYx?m=slm-PsQU4Tk5u9vp57lib2yu	This study
**pRV355 LidΔ6**	https://benchling.com/s/seq-DEOl5Zd86B486Mi5vjs9?m=slm-SVyRzIyKI7BlNu38DAx4	This study
**pRV356 LidΔ5**	https://benchling.com/s/seq-0sy0CxDyfwJLzV6rc7mm?m=slm-YGzzjjA0oOCGMjNWdBft	This study
**pRV357 LidΔ4**	https://benchling.com/s/seq-mQ53dbGtNSriVnT2MIe0?m=slm-cTucNmJcppH8ja9sYirn	This study
**pRV358 LidΔ3**	https://benchling.com/s/seq-70jEHOESN2XaaFhpGHfH?m=slm-MwXIGNy5jGP88e4C5C22	This study
**pRV359 LidΔ2**	https://benchling.com/s/seq-qS0X8lMvUniIBvhaq0Lw?m=slm-XrvLVVLBYGlNVheUBa9j	This study
**pRV360 LidΔ1**	https://benchling.com/s/seq-4TCB6l7bShZujJf3Ta38?m=slm-NFqmlM2NsnsS5Oc9XdRa	This study
**pRV361 LidΔ0**	https://benchling.com/s/seq-L7Vc5XSzhiIMSmhGjixg?m=slm-OdmyXABNHXcoOp8e7paY	This study
**pRV362 Lid2Δ3**	https://benchling.com/s/seq-gYT5bHevVRTurNR1wENV?	This study

	m=slm-t95vr5ADJbuPn8Lo9EEP	
**pRV363 Lid2Δ2**	https://benchling.com/s/seq-KcVwe7hWTq6mh5L8YWjf?m=slm-O8XXTgY1b9tDS0S8hbzx	This study
**pRV364 Lid2Δ1**	https://benchling.com/s/seq-Yk6DDPtjOOX2JsaqmJUn?m=slm-a8YQexhzPwYRJF3bS6dA	This study
**pRV365 Lid2Δ0**	https://benchling.com/s/seq-ERZQyhcaFMDau85lLbtq?m=slm-tcMAfw1bJdhjMjM9pM0C	This study
**pRV366 LidΔ11-N**	https://benchling.com/s/seq-hMcb0FDqrS0i2lsEU2NO?m=slm-93L6GOw87UTYRgJP1wnx	This study
**pRV367 LidΔ10-N**	https://benchling.com/s/seq-EnqTIAzvGHHkmwIahtz0?m=slm-y4Z8KjuatGxshowGlkKy	This study
**pRV368 LidΔ9-N**	https://benchling.com/s/seq-yKfb9xZYghWaeP4jkDql?m=slm-NzsyGFKo1zjb6eYMJNu7	This study
**pRV369 LidΔ8-N**	https://benchling.com/s/seq-kMuyC7XnA537wLeiV6Ob?m=slm-mLZ6xJ4i3FsU2Izn6qoB	This study
**pRV370 LidΔ7-N**	https://benchling.com/s/seq-zKnukWEPEMnPJBZXDP71?m=slm-BRgkEnMKMMgpqtS0x00T	This study
**pRV371 LidΔ6-N**	https://benchling.com/s/seq-TbsB7Wfb8C05MypIKk5F?m=slm-hwmzU6c2ScHD5p1zLFfd	This study
**pRV372 LidΔ5-N**	https://benchling.com/s/seq-gUVJcPVu1jhWQ330w5oQ?m=slm-W5fdcQkdjBnUWzXwWN7K	This study
**pRV373 LidΔ4-N**	https://benchling.com/s/seq-2LkysA90guExWzJHb88V?m=slm-FedC8MoU3ebegyUgsh18	This study
**pRV374 LidΔ3-N**	https://benchling.com/s/seq-wKLhiDPnGzwJS9C63kE9?m=slm-v17hL11USdhp2mR0XOOt	This study
**pRV375 LidΔ2-N**	https://benchling.com/s/seq-	This

	suE7swaqoHDLkGqCjFUk?m=slm-wtQ9MczfCH2JqiNtvT6I	study
**pRV376 LidΔ1-N**	https://benchling.com/s/seq-cpA6lFwV9WMdfaGvEdfO?m=slm-I698cqYCH6abPA5BpiZ3	This study
**pRV377 LidΔ0-N**	https://benchling.com/s/seq-87VLk6kiXIErPu2rfpfl?m=slm-SvlFphlSyzIIXra1w0JK	This study

Supplementary Table S3: Relevant sequences used in this study

Name	Seq	Note
**PS1**	TTTAACGATGGGAAACCTTACCCT	pRV041 (pGFP)
**PS2**	TTTACTCCACTGAACCTGGGGAAT	pRV046 (pRFP)
**Guide PS1 in vivo**	GTTTCAAAGATTAAATAATTTCTACTAAGTGTAGATACGA<br>TGGGAAACCTTACCCT	pRV041 (pGFP)
**Guide PS2**	GTTTCAAAGATTAAATAATTTCTACTAAGTGTAGATCTCC<br>ACTGAACCTGGGGAAT	pRV046 (pRFP)
**Target1 in vivo**	TTTAGTCGAGTGCAAAACCTTTCG	pRV013
**crRNA T**	UAAUUUCUACUAAGUGUAGAUGUCGAGUGCAAAACC<br>UUUCG	BG32778
**crRNA NT**	UAAUUUCUACUAAGUGUAGAUUGGUCUUCGCAUCUU<br>GCCGU	BG32779
**Expression vector LbCas12a wt (pET)**	MGSSHHHHHHENLYFQSNASKLEKFTNCYSLSKTLRFKAIP<br>VGKTQENIDNKRLLVEDEKRAEDYKGVKKLLDRYYLSFINDV<br>LHSIKLKNLNNYISLFRKKTRTEKENKELENLEINLRKEIAKAF<br>KGNEGYKSLFKKDIIETILPEFLDDKDEIALVNSFNGFTTAFTG<br>FFDNRENMFSEEAKSTSIAFRCINENLTRYISNMDIFEKVDAI<br>FDKHEVQEIKEKILNSDYDVEDFFEGEFFNFVLTQEGIDVYN<br>AIIGGFVTESGEKIKGLNEYINLYNQKTKQKLPKFKPLYKQVL<br>SDRESLSFYGEGYTSDEEVLEVFRNTLNKNSEIFSSIKKLEKLF<br>KNFDEYSSAGIFVKNGPAISTISKDIFGEWNVIRDKWNAEYD<br>DIHLKKKAVVTEKYEDDRRKSFKKIGSFSLEQLQEYADADLS<br>VVEKLKEIIIQKVDEIYKVYGSSEKLFDADFVLEKSLKKNDAVV<br>AIMKDLLDSVKSFENYIKAFFGEGKETNRDESFYGDFVLAYD<br>ILLKVDHIYDAIRNYVTQKPYSKDKFKLYFQNPQFMGGWDK<br>DKETDYRATILRYGSKYYLAIMDKKYAKCLQKIDKDDVNGN<br>YEKINYKLLPGPNKMLPKVFFSKKWMAYYNPSEDIQKIYKN<br>GTFKKGDMFNLNDCHKLIDFFKDSISRYPKWSNAYDFNFSE<br>TEKYKDIAGFYREVEEQGYKVSFESASKKEVDKLVEEGKLYM<br>FQIYNKDFSDKSHGTPNLHTMYFKLLFDENNHGQIRLSGGA<br>ELFMRRASLKKEELVVHPANSPIANKNPDNPKKTTTLSYDV<br>YKDKRFSEDQYELHIPIAINKCPKNIFKINTEVRVLLKHDDNP<br>YVIGIDRGERNLLYIVVVDGKGNIVEQYSLNEIINNFNGIRIKT<br>DYHSLLDKKEKERFEARQNWTSIENIKELKAGYISQVVHKIC<br>ELVEKYDAVIALEDLNSGFKNSRVKVEKQVYQKFEKMLIDKL<br>NYMVDKKSNPCATGGALKGYQITNKFESFKSMSTQNGFIF<br>YIPAWLTSKIDPSTGFVNLLKTKYTSIADSKKFISSFDRIMYVP<br>EEDLFEFALDYKNFSRTDADYIKKWKLYSYGNRIRIFRNPKK<br>NNVFDWEEVCLTSAYKELFNKYGINYQQGDIRALLCEQSDK<br>AFYSSFMALMSLMLQMRNSITGRTDVDFLISPVKNSDGIFY<br>DSRNYEAQENAILPKNADANGAYNIARKVLWAIGQFKKAE<br>DEKLDKVKIAISNKEWLEYAQTSVK*	pRV020
**Expression vector**	MGSSHHHHHHENLYFQSNASKLEKFTNCYSLSKTLRFKAIP	pET ΔLid2

**LbCas12a Lid2 (pET)**	VGKTQENIDNKRLLVEDEKRAEDYKGVKKLLDRYYLSFINDV<br>LHSIKLKNLNNYISLFRKKTRTEKENKELENLEINLRKEIAKAF<br>KGNEGYKSLFKKDIIETILPEFLDDKDEIALVNSFNGFTTAFTG<br>FFDNRENMFSEEAKSTSIAFRCINENLTRYISNMDIFEKVDAI<br>FDKHEVQEIKEKILNSDYDVEDFFEGEFFNFVLTQEGIDVYN<br>AIIGGFVTESGEKIKGLNEYINLYNQKTKQKLPKFKPLYKQVL<br>SDRESLSFYGEGYTSDEEVLEVFRNTLNKNSEIFSSIKKLEKLF<br>KNFDEYSSAGIFVKNGPAISTISKDIFGEWNVIRDKWNAEYD<br>DIHLKKKAVVTEKYEDDRRKSFKKIGSFSLEQLQEYADADLS<br>VVEKLKEIIIQKVDEIYKVYGSSEKLFDADFVLEKSLKKNDAVV<br>AIMKDLLDSVKSFENYIKAFFGEGKETNRDESFYGDFVLAYD<br>ILLKVDHIYDAIRNYVTQKPYSKDKFKLYFQNPQFMGGWDK<br>DKETDYRATILRYGSKYYLAIMDKKYAKCLQKIDKDDVNGN<br>YEKINYKLLPGPNKMLPKVFFSKKWMAYYNPSEDIQKIYKN<br>GTFKKGDMFNLNDCHKLIDFFKDSISRYPKWSNAYDFNFSE<br>TEKYKDIAGFYREVEEQGYKVSFESASKKEVDKLVEEGKLYM<br>FQIYNKDFSDKSHGTPNLHTMYFKLLFDENNHGQIRLSGGA<br>ELFMRRASLKKEELVVHPANSPIANKNPDNPKKTTTLSYDV<br>YKDKRFSEDQYELHIPIAINKCPKNIFKINTEVRVLLKHDDNP<br>YVIGIDRGERNLLYIVVVDGKGNIVEQYSLNEIINNFNGIRIKT<br>DYHSLLDKKEKERFEARQNWTSIENIKELKAGYISQVVHKIC<br>ELVEKYDAVIAL**E**DLNSYCSSVYQKFEKMLIDKLNYMVDKKS<br>NPCATGGALKGYQITNKFESFKSMSTQNGFIFYIPAWLTSKI<br>DPSTGFVNLLKTKYTSIADSKKFISSFDRIMYVPEEDLFEFALD<br>YKNFSRTDADYIKKWKLYSYGNRIRIFRNPKKNNVFDWEEV<br>CLTSAYKELFNKYGINYQQGDIRALLCEQSDKAFYSSFMAL<br>MSLMLQMRNSITGRTDVDFLISPVKNSDGIFYDSRNYEAQE<br>NAILPKNA**D**ANGAYNIARKVLWAIGQFKKAEDEKLDKVKIA<br>ISNKEWLEYAQTSVK*	
**LbCas12a wt (pCas)**	MSKLEKFTNCYSLSKTLRFKAIPVGKTQENIDNKRLLVEDEK<br>RAEDYKGVKKLLDRYYLSFINDVLHSIKLKNLNNYISLFRKKTR<br>TEKENKELENLEINLRKEIAKAFKGNEGYKSLFKKDIIETILPEF<br>LDDKDEIALVNSFNGFTTAFTGFFDNRENMFSEEAKSTSIAF<br>RCINENLTRYISNMDIFEKVDAIFDKHEVQEIKEKILNSDYDV<br>EDFFEGEFFNFVLTQEGIDVYNAIIGGFVTESGEKIKGLNEYI<br>NLYNQKTKQKLPKFKPLYKQVLSDRESLSFYGEGYTSDEEVL<br>EVFRNTLNKNSEIFSSIKKLEKLFKNFDEYSSAGIFVKNGPAIS<br>TISKDIFGEWNVIRDKWNAEYDDIHLKKKAVVTEKYEDDRR<br>KSFKKIGSFSLEQLQEYADADLSVVEKLKEIIIQKVDEIYKVYG<br>SSEKLFDADFVLEKSLKKNDAVVAIMKDLLDSVKSFENYIKA<br>FFGEGKETNRDESFYGDFVLAYDILLKVDHIYDAIRNYVTQK<br>PYSKDKFKLYFQNPQFMGGWDKDKETDYRATILRYGSKYYL<br>AIMDKKYAKCLQKIDKDDVNGNYEKINYKLLPGPNKMLPKV<br>FFSKKWMAYYNPSEDIQKIYKNGTFKKGDMFNLNDCHKLI<br>DFFKDSISRYPKWSNAYDFNFSETEKYKDIAGFYREVEEQGY<br>KVSFESASKKEVDKLVEEGKLYMFQIYNKDFSDKSHGTPNL<br>HTMYFKLLFDENNHGQIRLSGGAELFMRRASLKKEELVVHP	pRV060

	ANSPIANKNPDNPKKTTTLSYDVYKDKRFSEDQYELHIPIAIN<br>KCPKNIFKINTEVRVLLKHDDNPYVIGIDRGERNLLYIVVVDG<br>KGNIVEQYSLNEIINNFNGIRIKTDYHSLLDKKEKERFEARQN<br>WTSIENIKELKAGYISQVVHKICELVEKYDAVIAL**E**DLNS**GFK**<br>**NSRVKVEK**QVYQKFEKMLIDKLNYMVDKKSNPCATGGAL<br>KGYQITNKFESFKSMSTQNGFIFYIPAWLTSKIDPSTGFVNLL<br>KTKYTSIADSKKFISSFDRIMYVPEEDLFEFALDYKNFSRTDA<br>DYIKKWKLYSYGNRIRIFRNPKKNNVFDWEEVCLTSAYKELF<br>NKYGINYQQGDIRALLCEQSDKAFYSSFMALMSLMLQMR<br>NSITGRTDVDFLISPVKNSDGIFYDSRNYEAQENAILPKNA**D**<br>ANGAYNIARKVLWAIGQFKKAEDEKLDKVKIAISNKEWLEY<br>AQTSVK*	
**LbCas12a dead D832A/E925A**	MSKLEKFTNCYSLSKTLRFKAIPVGKTQENIDNKRLLVEDEK<br>RAEDYKGVKKLLDRYYLSFINDVLHSIKLKNLNNYISLFRKKTR<br>TEKENKELENLEINLRKEIAKAFKGNEGYKSLFKKDIIETILPEF<br>LDDKDEIALVNSFNGFTTAFTGFFDNRENMFSEEAKSTSIAF<br>RCINENLTRYISNMDIFEKVDAIFDKHEVQEIKEKILNSDYDV<br>EDFFEGEFFNFVLTQEGIDVYNAIIGGFVTESGEKIKGLNEYI<br>NLYNQKTKQKLPKFKPLYKQVLSDRESLSFYGEGYTSDEEVL<br>EVFRNTLNKNSEIFSSIKKLEKLFKNFDEYSSAGIFVKNGPAIS<br>TISKDIFGEWNVIRDKWNAEYDDIHLKKKAVVTEKYEDDRR<br>KSFKKIGSFSLEQLQEYADADLSVVEKLKEIIIQKVDEIYKVYG<br>SSEKLFDADFVLEKSLKKNDAVVAIMKDLLDSVKSFENYIKA<br>FFGEGKETNRDESFYGDFVLAYDILLKVDHIYDAIRNYVTQK<br>PYSKDKFKLYFQNPQFMGGWDKDKETDYRATILRYGSKYYL<br>AIMDKKYAKCLQKIDKDDVNGNYEKINYKLLPGPNKMLPKV<br>FFSKKWMAYYNPSEDIQKIYKNGTFKKGDMFNLNDCHKLI<br>DFFKDSISRYPKWSNAYDFNFSETEKYKDIAGFYREVEEQGY<br>KVSFESASKKEVDKLVEEGKLYMFQIYNKDFSDKSHGTPNL<br>HTMYFKLLFDENNHGQIRLSGGAELFMRRASLKKEELVVHP<br>ANSPIANKNPDNPKKTTTLSYDVYKDKRFSEDQYELHIPIAIN<br>KCPKNIFKINTEVRVLLKHDDNPYVIGIARGERNLLYIVVVDG<br>KGNIVEQYSLNEIINNFNGIRIKTDYHSLLDKKEKERFEARQN<br>WTSIENIKELKAGYISQVVHKICELVEKYDAVIALADLNSGFK<br>NSRVKVEKQVYQKFEKMLIDKLNYMVDKKSNPCATGGALK<br>GYQITNKFESFKSMSTQNGFIFYIPAWLTSKIDPSTGFVNLLK<br>TKYTSIADSKKFISSFDRIMYVPEEDLFEFALDYKNFSRTDAD<br>YIKKWKLYSYGNRIRIFRNPKKNNVFDWEEVCLTSAYKELFN<br>KYGINYQQGDIRALLCEQSDKAFYSSFMALMSLMLQMRNS<br>ITGRTDVDFLISPVKNSDGIFYDSRNYEAQENAILPKNADAN<br>GAYNIARKVLWAIGQFKKAEDEKLDKVKIAISNKEWLEYAQ<br>TSVK*	pRV061
**LbCas12a ΔLid2**	MSKLEKFTNCYSLSKTLRFKAIPVGKTQENIDNKRLLVEDEK<br>RAEDYKGVKKLLDRYYLSFINDVLHSIKLKNLNNYISLFRKKTR<br>TEKENKELENLEINLRKEIAKAFKGNEGYKSLFKKDIIETILPEF<br>LDDKDEIALVNSFNGFTTAFTGFFDNRENMFSEEAKSTSIAF<br>RCINENLTRYISNMDIFEKVDAIFDKHEVQEIKEKILNSDYDV	pRV25836<br>-Lid2 pCas

EDFFEGEFFNFVLTQEGIDVYNAIIGGFVTESGEKIKGLNEYI
NLYNQKTKQKLPKFKPLYKQVLSDRESLSFYGEGYTSDEEVL
EVFRNTLNKNSEIFSSIKKLEKLFKNFDEYSSAGIFVKNGPAIS
TISKDIFGEWNVIRDKWNAEYDDIHLKKKAVVTEKYEDDRR
KSFKKIGSFSLEQLQEYADADLSVVEKLKEIIIQKVDEIYKVYG
SSEKLFDADFVLEKSLKKNDAVVAIMKDLLDSVKSFENYIKA
FFGEGKETNRDESFYGDFVLAYDILLKVDHIYDAIRNYVTQK
PYSKDKFKLYFQNPQFMGGWDKDKETDYRATILRYGSKYYL
AIMDKKYAKCLQKIDKDDVNGNYEKINYKLLPGPNKMLPKV
FFSKKWMAYYNPSEDIQKIYKNGTFKKGDMFNLNDCHKLI
DFFKDSISRYPKWSNAYDFNFSETEKYKDIAGFYREVEEQGY
KVSFESASKKEVDKLVEEGKLYMFQIYNKDFSDKSHGTPNL
HTMYFKLLFDENNHGQIRLSGGAELFMRRASLKKEELVVHP
ANSPIANKNPDNPKKTTTLSYDVYKDKRFSEDQYELHIPIAIN
KCPKNIFKINTEVRVLLKHDDNPYVIGIDRGERNLLYIVVVDG
KGNIVEQYSLNEIINNFNGIRIKTDYHSLLDKKEKERFEARQN
WTSIENIKELKAGYISQVVHKICELVEKYDAVIAL**E**DLNS**YCS**
SVYQKFEKMLIDKLNYMVDKKSNPCATGGALKGYQITNKFE
SFKSMSTQNGFIFYIPAWLTSKIDPSTGFVNLLKTKYTSIADS
KKFISSFDRIMYVPEEDLFEFALDYKNFSRTDADYIKKWKLYS
YGNRIRIFRNPKKNNVFDWEEVCLTSAYKELFNKYGINYQQ
GDIRALLCEQSDKAFYSSFMALMSLMLQMRNSITGRTDVD
FLISPVKNSDGIFYDSRNYEAQENAILPKNA**D**ANGAYNIARK
VLWAIGQFKKAEDEKLDKVKIAISNKEWLEYAQTSVK*

Supplementary Table S4. Data collection, processing, model refinement, and validation.

**Data collection and processing**	
**Sample**	**LbCas12a-ΔLid2–crRNA–DNA complex**
**EMDB ID**	**EMD-66115**
**PDB ID**	**9WNE**
**Microscope**	**Titan Krios G3i**
**Detector**	**Gatan K3 Camera**
**Magnification**	**105**
**Voltage (kV)**	**300**
**Electron exposure (e–/Å2)**	**50**
**Defocus range (μm)**	**−0.8 to −1.6**
**Pixel size (Å)**	**0.83**
**Symmetry imposed**	**C1**
**Number of movies**	**5,403**
**Initial particle images (no.)**	**7,190,848**
**Final particle images (no.)**	**100,683**
**Map resolution (Å)**	**2.54**
**FSC threshold**	**0.143**
**3DFSC analysis**	
**Global resolution (Å)**	**2.69**
**Sphericity**	**0.888**
**Model building and refinement**	
**Map sharpening B factor (Å2)**	**−60.6**
**Model composition**	
**Protein atoms**	**10,068**
**Nucleic acid atoms**	**1,889**
**Other atoms**	**23**
**Model refinement**	
**Resolution (Å) by model to map FSC, threshold 0.50**	**2.54**
**Average B factor (Å2) (protein/nucleotides/Ligand /Water)**	**71.5/ 35.2/ 66.7/ 37.7**
**R.m.s. deviations**	
**Bond lengths (Å)**	**0.0088**
**Bond angles (°)**	**1.5**
**Validation**	
**MolProbity score**	**1.26**
**Clashscore**	**2.13**
**Rotamer outliers (%)**	**0.36**
**Cβ outliers (%)**	**0.17**

**EMRinger score**	**4.49**
**Ramachandran plot**	
**Favored (%)**	**95.97**
**Allowed (%)**	**4.03**
**Outliers (%)**	**0.00**

Supplementary Table S5. Engineered Lid variants: Lid sequence and nicking phenotype.
Lid amino-acid sequence (positions S929 to Q941; dashes are deletions relative to the wild-type Lid) and the qualitative reporter phenotype for every engineered Lid variant in this study, grouped by engineering series. The trimming series (Lid1Δ-C, Lid1Δ-N, Lid2Δ) is included; its per-variant reporter readouts are shown in main Figure 3B, Figure 3D and Supplementary Figure 3.

Series	Variant	Lid (S929-Q941)
wild type	WT	SGFKNSRVKVEKQ
rational design (Lid)	K932G/N933G	SGFGGSRVKVEKQ
	S934A/R935G	SGFKNAGVKVEKQ
	K932G/N933G/S934A/R935G	SGFGGAGVKVEKQ
	ΔE939/K940G	SGFKNSRVKV-GQ
	Lid neutral	SGGAGSGVAVGGQ
	Lid negative	SGEEGSDVDVEDQ
	Lid positive	SGRKNKRVKVRKQ
rational design (RRM grafts)	RRM-1	SGEKESGVSVGGQ
	RRM-2	SGSKGSGVNVNGQ
	RRM-3	SGESRSKVRVDNQ
rational design (proline)	Q888P/N889P	SGFKNSRVKVEKQ
NNK library (Lid1)	Lid1.1	SGSINKWRYPEAQ
	Lid1.2	SGKFSFLVSSSKQ
	Lid1.3	SGIIMLKHASGCQ
	Lid1.4	SGYWLHRQFTGRQ
	Lid1.5	SGFKNKWRYPEAQ
	Lid1.6	SGFKNFLVSSSKQ
	Lid1.7	SGLLFWSCNGGLQ
	Lid1.8	SGFKNWSCNGGLQ
	Lid1.9	SGKKNFLVSSSKQ
Lid2 artifact	Lid2 (YCSS)	SYCSS--------
Lid1Δ trimming series	Lid1Δ1-C	SGFKNSRVKVEK-
	Lid1Δ2-C	SGFKNSRVKVE--
	Lid1Δ3-C	SGFKNSRVKV---
	Lid1Δ4-C	SGFKNSRVK----
	Lid1Δ5-C	SGFKNSRV-----
	Lid1Δ6-C	SGFKNSR------
	Lid1Δ7-C	SGFKNS-------
	Lid1Δ8-C	SGFKN--------
	Lid1Δ9-C	SGFK---------
	Lid1Δ10-C	SGF----------
	Lid1Δ11-C	SG-----------
	Lid1Δ12-C	S------------
	Lid2Δ1-C	SYCS---------
	Lid2Δ2-C	SYC----------
	Lid2Δ3-C	SY-----------

	Lid2Δ4-C	S-----------
	Lid1Δ1-N	S-FKNSRVKVEKQ
	Lid1Δ2-N	S--KNSRVKVEKQ
	Lid1Δ3-N	S---NSRVKVEKQ
	Lid1Δ4-N	S----SRVKVEKQ
	Lid1Δ5-N	S-----RVKVEKQ
	Lid1Δ6-N	S------VKVEKQ
	Lid1Δ7-N	S-------KVEKQ
	Lid1Δ8-N	S--------VEKQ
	Lid1Δ9-N	S---------EKQ
	Lid1Δ10-N	S----------KQ
	Lid1Δ11-N	S-----------Q
	Lid1Δ12-N	S------------
Lid4 sweet spot	Lid2.2 (CLRR)	SCLRR--------
	Lid2.3 (YLGT)	SYLGT--------
	Lid2.4 (PITR)	SPITR--------
	Lid2.5 (IFRR)	SIFRR--------
	Lid2.6 (PRSS)	SPRSS--------
	Lid2.7 (YIRR)	SYIRR--------
	Lid2.8 (TMFR)	STMFR--------

Supplementary Table S6. Oligonucleotides used in the lid trimming assay.

Name	Sequence	BG code
Lid1Δ1-C fw	ggctttaaaaacagccgggtgaaggtggagaaagtataccaaaagtttgaaaagatgc	BG33922
Lid1Δ2-C fw	ggctttaaaaacagccgggtgaaggtggaggtataccaaaagtttgaaaagatgc	BG33923
Lid1Δ3-C fw	ggctttaaaaacagccgggtgaaggtggtataccaaaagtttgaaaagatgc	BG33924
Lid1Δ4-C fw	ggctttaaaaacagccgggtgaaggtataccaaaagtttgaaaagatgc	BG33925
Lid1Δ5-C fw	ggctttaaaaacagccgggtggtataccaaaagtttgaaaagatgc	BG33926
Lid1Δ6-C fw	ggctttaaaaacagccgggtataccaaaagtttgaaaagatgc	BG33927
Lid1Δ7-C fw	ggctttaaaaacagcgtataccaaaagtttgaaaagatgc	BG33928
Lid1Δ8-C fw	ggctttaaaaacgtataccaaaagtttgaaaagatgc	BG33929
Lid1Δ9-C fw	ggctttaaagtataccaaaagtttgaaaagatgc	BG33930
Lid1Δ10-C fw	ggctttgtataccaaaagtttgaaaagatgc	BG33931
Lid1Δ11-C fw	ggcgtataccaaaagtttgaaaagatgc	BG33932
Lid1Δ12-C fw	gtataccaaaagtttgaaaagatgc	BG33933
Lid2Δ1-C fw	TACTGCTCcgtataccaaaagtttgaaaagatgctcattg	BG33935
Lid2Δ2-C fw	TACTGCgtataccaaaagtttgaaaagatgctcattg	BG33936
Lid2Δ3-C fw	TACgtataccaaaagtttgaaaagatgctcattg	BG33937
Lid2Δ4-C fw	gtataccaaaagtttgaaaagatgctcattg	BG33938
Lid C-to-N Rv common	gctattgaggtcttccagg	BG33965
Lid1Δ1-N fw	tttaaaaacagccgggtgaaggtggagaaacaggtataccaaaagtttgaaaagatgc	BG34180
Lid1Δ2-N fw	aaaaacagccgggtgaaggtggagaaacaggtataccaaaagtttgaaaagatgc	BG34181
Lid1Δ3-N fw	aacagccgggtgaaggtggagaaacaggtataccaaaagtttgaaaagatgc	BG34182
Lid1Δ4-N fw	agccgggtgaaggtggagaaacaggtataccaaaagtttgaaaagatgc	BG34183
Lid1Δ5-N	cgggtgaaggtggagaaacaggtataccaaaagtttgaaaagatgc	BG34184

fw		
Lid1Δ6-N fw	gtgaaggtggagaaacaggtataccaaaagtttgaaaagatgc	BG34185
Lid1Δ7-N fw	aaggtggagaaacaggtataccaaaagtttgaaaagatgc	BG34186
Lid1Δ8-N fw	gtggagaaacaggtataccaaaagtttgaaaagatgc	BG34187
Lid1Δ9-N fw	gagaaacaggtataccaaaagtttgaaaagatgc	BG34188
Lid1Δ10-N fw	aaacaggtataccaaaagtttgaaaagatgc	BG34189
Lid1Δ11-N fw	caggtataccaaaagtttgaaaagatgc	BG34190
Lid1Δ12-N fw	gtataccaaaagtttgaaaagatgc	BG34191
Lid N-to-C Rv common	gctattgaggtcttccagg	BG33965

Supplementary Table S7. Per-position Sanger base calls and base-call quality across the LbCas12a-Lid2 non-target-strand cleavage site.

Pos	NTS A	NTS C	NTS G	NTS T	NTS call	NTS Q	TS A	TS C	TS G	TS T	TS call	TS Q
PAM	0	0	0	2	T	23	0	0	0	601	T	61
PAM	0	0	0	3	T	34	0	0	0	575	T	58
PAM	0	0	0	2	T	34	0	0	0	519	T	61
PAM	3	0	0	0	A	39	545	0	0	0	A	58
1	0	0	3	0	G	43	0	0	521	0	G	58
2	0	0	0	4	T	46	0	0	0	583	T	58
3	0	1	0	0	G	18	0	634	0	0	C	61
4	0	0	4	0	G	48	0	0	407	0	G	58
5	4	0	0	0	A	46	673	0	1	0	A	58
6	0	0	4	0	G	49	0	0	430	0	G	58
7	0	0	0	2	T	30	0	0	3	560	T	58
8	0	0	4	0	G	49	0	0	606	0	G	61
9	0	4	0	0	C	34	0	514	0	0	C	61
10	4	0	0	0	A	36	477	0	0	0	A	61
11	3	0	0	0	A	28	508	0	0	0	A	61
12	4	0	0	0	A	11	513	0	0	0	A	61
13	0	11	0	33	T	14	531	0	0	0	A	61
**14**	0	409	0	18	C	59	0	488	0	0	C	51
15	1	7	0	916	T	59	0	606	0	0	C	51
16	3	3	0	939	T	56	0	0	0	580	T	58
17	3	11	0	1053	T	49	0	0	0	640	T	61
18	0	891	6	44	C	59	0	0	0	581	T	61
19	0	13	977	0	G	62	0	606	0	0	C	61
20	3	958	29	17	C	62	0	2	472	0	G	61
21	0	6	980	9	G	62	0	568	3	0	C	61
22	4	0	930	2	G	62	0	0	618	0	G	61
23	5	2	2	789	T	49	0	0	602	0	G	58
24	635	0	5	22	A	35	0	0	0	410	T	51
25	2	1	0	641	T	12	660	0	0	1	A	58
**26**	1	4	903	0	G	46	0	0	0	356	T	51
27	3	0	749	0	G	62	0	0	654	0	G	61
28	12	982	6	1	C	62	0	0	583	0	G	61
29	1012	13	1	2	A	62	0	517	0	0	C	61
30	1034	8	0	6	A	62	506	0	0	0	A	61

The table reports, for each nucleotide position downstream of the TTTA PAM, the raw ABIF trace amplitude in all four base channels (A, C, G, T) at the called peak, the base call, and the Phred quality score, for the non-target strand (NTS; read 2178ZAG609) and the target strand (TS; read 2178ZAG608) of the in vitro cleaved pRV013 plasmid. Positions are numbered from the first nucleotide 3′ of the PAM; both reads are oriented to the (+) reference. Values are extracted directly from the primary AB1 files (PLOC1, PBAS1, PCON1, DATA9–12); the quality cell is shaded from green (high) to red (low) on the same scale used in the figure.

References

1. Zetsche B, Gootenberg JS, Abudayyeh OO, et al. Cpf1 Is a Single RNA-Guided Endonuclease of a Class 2 CRISPR-Cas System. *Cell*. 2015;163(3):759-771. doi:10.1016/j.cell.2015.09.038

2. Wu WY, Mohanraju P, Liao C, et al. The miniature CRISPR-Cas12m effector binds DNA to block transcription. *Molecular Cell*. 2022;82(23):4487-4502.e7. doi:10.1016/j.molcel.2022.11.003

3. Swarts DC, Van Der Oost J, Jinek M. Structural Basis for Guide RNA Processing and Seed-Dependent DNA Targeting by CRISPR-Cas12a. *Molecular Cell*. 2017;66(2):221-233.e4. doi:10.1016/j.molcel.2017.03.016
